# Two-photon AIE luminogen labeled multifunctional polymeric micelles for theranostics

**DOI:** 10.7150/thno.33901

**Published:** 2019-08-27

**Authors:** Weihua Zhuang, Boxuan Ma, Jun Hu, Jizhou Jiang, Gaocan Li, Li Yang, Yunbing Wang

**Affiliations:** National Engineering Research Center for Biomaterials, Sichuan University, 29 Wangjiang Road, Chengdu 610064, China.

**Keywords:** theranostics, aggregation induced emission, drug delivery, two-photon, bioimaging.

## Abstract

Intelligent polymeric micelles with fluorescence imaging feature have been emerged as promising tools for theranostics. However, conventional fluorescent dyes are limited by short wavelength excitation, interference of tissue autofluorescence, limited imaging depth and quenched emission in aggregation state.

**Methods**: We synthesized a novel mPEG-SS-Poly (AEMA-*co*-TBIS) (mPEATss) copolymer to develop multifunctional polymeric micelles with great AIE feature for cancer therapy and AIE active two-photon bioimaging. The stimuli-responsive behavior and AIE active two-photon cell and tissue imaging as well as *in vitro* and *in vivo* antitumor ability of DOX-loaded mPEATss were studied.

**Results**: mPEATss micelles showed excellent AIE active two-photon cell imaging ability and deep tissue imaging ability. Antitumor drug DOX could be encapsulated to form a drug-loaded micellar system with a small diameter of 65 nm. The disassembly and charge-conversion of mPEATss micelles could be triggered by acidic environment, resulting in accelerated drug release and great antitumor efficacy. *In vivo*, *ex vivo* imaging and *in vivo* pharmacokinetic study demonstrated that mPEATss micelles could efficiently accumulate in tumor sites, which ensured ideal anticancer effect.

**Conclusions**: This pH and redox dual responsive and AIE active two-photon imaging polymeric micelles would be a promising candidate for theranostics.

## Introduction

Fluorescent compounds with aggregation-induced quenching (ACQ) feature are limited for bioimaging applications, whereas aggregation-induced emission (AIE) fluorescent compounds would be more promising fluorescent probes for bioimaging due to their high-efficiency, sensitivity and strong fluorescence in aggregation state 1-3. Conventional one-photon excited AIE molecules are usually excited by light irradiation of short wavelength, leading to cell and tissue toxicity, and their biomedical application is also limited by limited penetration depth (only a few tens of micrometers), interference of tissue autofluorescence and photobleaching phenomenon 2, 4-5. Therefore, fluorescence imaging with long wavelength excitation in the near-infrared (NIR) or infrared window is highly attractive due to its significant reduction in biological damage and tissue autofluorescence 4, 6.

In view of the great advantage of NIR excitation fluorescence imaging, two-photon fluorescence imaging (TPFI) has provided a powerful weapon for bioimaging with low-energy irradiation but high-energy fluorescence, which is featured with low biological damage, significant reduction of autofluorescence and scattering related to the longer exciting wavelengths and high signal noise ratio at a deep focal plane due to the great penetration depth 6-8. Moreover, the ultrahigh resolution of TPFI would make it more advantageous than MRI and CT in real-time monitoring. Although MRI and CT are frequently-used attributed to their superior penetration depth in cancer diagnosis, the problems of long acquisition time for both MRI and CT still remain 5, 9-11. Thus, development of novel two-photon fluorescent probes with aggregation-induced emission would be a great help for pinpointing tumor locations.

Thus far, there are many fluorophores reported, which exhibit great cellular imaging ability and would also be suitable for cancer diagnosis. However, their further application *in vivo* is often suffered from their poor solubility resulting in short half-life in the serum and low bioavailability of the fluorescence probes 11-14*.* In view of the great advantages of drug delivery systems (DDS) based on biocompatible macromolecules 15-16, which are marked with enhanced accumulation and long-circulating, the combination of DDS and TPFI would promote the biological application of two-photon fluorophores by precisely finding out the location of tumor via the enhanced permeability and retention (EPR) effect 17-18. In addition, theranostic nanomedicine, which combines DDS and cancer diagnosis, has become the center of attention in recent years 19-20. Searching for high-efficient AIE active two-photon fluorophores and novel DDS is in great demand and would promote the development of cancer diagnosis and cancer therapy.

Furthermore, in order to obtain accurate cancer diagnosis with simultaneously enhanced antitumor efficacy, it would be more desirable to combine environment triggered drug release with AIE active TPFI. The specific microenvironment of tumor makes it an available strategy for constructing stimuli triggered drug release, such as pH responsiveness 21-22 and redox responsiveness 23-24, which have been reported to improve antitumor efficacy. However, the antitumor effect of single pH response or redox response still needs ulteriorly improve 25-27. On the contrary, DDS with synergetic dual-response or multi-response have showed better antitumor effect, which would be suitable for designing smart DDS for theranostics 28-30.

In this work, we have developed novel polymeric micelles based on mPEG-SS-Poly (AEMA-*co*-TBIS) (mPEATss) copolymer with two-photon excitable AIE fluorescence probe equipped with pH and redox-dual triggered drug release as well as charge-conversion induced enhancement of cell internalization for theranostics. mPEATss micelles show great biocompatibility and desired AIE feature. Moreover, mPEATss micelles exhibit great sensitivity to acid and high concentration of glutathione (GSH) as well as charge-conversion under acidic condition. Antitumor drug doxorubicin (DOX) is encapsulated and the drug release can be triggered by both acid and GSH. In addition, the AIE active high-quality imaging is confirmed in cells and tissues. The antitumor efficacy of DOX-loaded micelles was further studied* in vitro* and *in vivo*, and the biodistribution of micelles is also evaluated. The excellent antitumor efficacy with reduced side effect and the great two-photon bioimaging of cells and tissues make these mPEATss micelles highly attractive to be a candidate for cancer theranostics.

## Materials and Methods

### Materials

Methoxy polyethylene glycol (mPEG, Mn = 5000) was obtained from Adamas Reagent, Ltd (Shanghai, China). 4-nitrophenyl chloroformate, azodiisobutyronitrile and cystamine dihydrochloride were purchased from Chengdu Best Reagent Co., LTD (Chengdu, China). Doxorubicin hydrochloride (DOX•HCl) was purchased from Dalian Meilun Biotechnology Co., LTD (Dalian, China). Tetrahydrofuran (THF), dichloromethane (DCM) and dimethyl formamide (DMF) were distilled under reduced pressure after dried over CaH_2_. All other chemicals were obtained from commercial supplier and used as received. 2-azepane ethyl methacrylate (AEMA) was synthesized according to previous report 30. The synthesis of TBisMA was shown in Supporting Information (Figure S1).

### Characterization

The ^1^H NMR spectra was recorded on a Bruker AV II-400. The molecular weight distribution was measured by an Agilent 1260 gel permeation chromatography (GPC) using THF as the eluent (flow rate: 1 mL/min at 40 ^o^C) and the calibration curve was obtained from a series of narrow PS standards. The size and zeta potential of micelles were measured by a Malvern Zetasizer Nano ZS at 25^ o^C. Transmission electron microscopy (TEM) images were obtained on a Hitachi H-600 transmission electron microscope. The AIE behavior was measured on a Hitachi fluorescence spectrophotometer F-4700.

### Synthesis of poly (AEMA-*co*-TBIS)

AIBN (37.40 mg, 0.23 mmol) TBISMA (1.0 g, 1.26 mmol), AEMA (1.6 g, 7.58 mmol) and 4-Cyanopentanoic acid dithiobenzoate (211.6 mg, 0.76 mmol) were dissolved in a Schlenk flask with 15 mL THF. After being degassed with three cycles of a freeze-pump-thaw procedure, the reaction was performed at 70 ^o^C under the protection of argon (Ar) for 24 h. The resulted solution was dialyzed (MCWO = 1000) against deionized water for 36 h, followed by freeze-drying to obtain poly (AEMA-*co*-TBIS) (2.48 g, yield 95.39%).

### Synthesis of mPEG-SS-NH_2_

The terminal hydroxyl group of mPEG-OH was activated by 4-nitrophenyl chloroformate. Briefly, mPEG-OH (10 g, 2 mmol) was totally dissolved in 200 mL dry THF with triethylamine (4.2 mL, 30 mmol). 4-Nitrophenyl chloroformate (4.03 g, 20 mmol) dissolved in 30 mL dry THF was added dropwise into the stirring mixed solution in ice bath. The reaction was performed at 50 ^o^C for 48 h. After that, the solution was filtered and concentrated by rotary evaporation and precipitated into excess amount of cold ethyl ether, which was repeated for five times to remove the excess 4-nitrophenyl chloroformate. mPEG-NO_2_ was obtained by vacuum drying at room temperature for 24 h (8.2 g, yield 79.38%).

The hydrochloride of cystamine dihydrochloride was removed according to previous work 31. Under Ar atmosphere, mPEG-NO_2_ (6 g, 1.16 mmol) was totally dissolved in 50 mL dry DCM. The solution was added into stirring cystamine (1.827 g, 11.6 mmol) solution (10 mL DCM), and the resulting solution was allowed to stir for 30 h at room temperature. The solution was concentrated and redissolved in THF. Afterwards, the mixture was dialyzed against deionized water (MWCO = 2000) for 2 days. mPEG-SS-NH_2_ was obtained by freeze-drying (5.6 g, yield 92.78%).

### Synthesis of mPEG-SS-Poly (AEMA-*co*-TBIS) and mPEG-Poly (AEMA-*co*-TBIS)

Under Ar atmosphere, poly (AEMA-*co*-TBIS) (0.50 g, 0.15 mmol) was dissolved in a mixture of 15 mL dry THF and 5 mL dry DMF. Afterwards, EDC·HCl (37.95 mg, 0.17 mmol) and NHS (19.18 mg, 0.17 mmol) were added, and the mixed solution was stirred at room temperature for 24 h. Under Ar atmosphere, mPEG-SS-NH_2_ (784.85 mg, 0.15 mmol) was dissolved in 10 mL dry THF and the solvent of poly (AEMA-*co*-TBIS) was added dropwise into it. The reaction was carried out for another 48 h at room temperature. The solution was concentrated under vacuum and dialyzed against deionized water (MWCO = 5000) for 48 h, followed by centrifugation (4000 rpm) to remove the precipitate and mPEG-SS-Poly (AEMA-*co*-TBIS) was obtained by freeze-drying (0.64 g, yield 47.5%). mPEG-Poly (AEMA-*co*-TBIS) (mPEAT) was synthesized in the same way (yield 49.1%).

### Preparation of DOX-loaded micelles and blank micelles

Typically, 20 mg copolymer, 4 mg DOX·HCl and 4 μL triethylamine were dissolved in 2 mL mixed solution of THF and DMF (1 : 1) and the solution was stirred at room temperature for 1 h. Afterwards, the solution was quickly injected into 6 mL deionized water (pH 7.4), and the mixed solution was further stirred at room temperature for 2 h. The resulted solution was dialyzed against deionized water (pH 7.4) for 24 h to remove the solvent and the unencapsulated drug. The blank micelles were prepared in the same way without the addition of DOX.

### Stimuli-responsive of micelles and *in vitro* drug release

The stimuli-responsive of mPEATss micelles and mPEAT micelles were carried out by incubating micelles at medium with or without 10 mM GSH at pH 7.4 or 6.0, and the changes of particle size were monitored by DLS.

The *in vitro* drug release of mPEATss and mPEAT micelles were performed in PBS (pH = 6.0 or 7.4) with or without 10 mM GSH at a DOX-loaded micelles concentration of 1 mg/mL. The micelle solution was transferred to a dialysis tube (MWCO = 3500) and the tube were immersed in 40 mL release medium. Afterwards, the whole drug release process was allowed to keep in the dark and continuous shaking. At preselected time interval, 2 mL release medium was taken out and 2 mL fresh medium was added. The amount of drug release was determined by fluorescence spectra, and the accumulative drug release was calculated.

### Cell culture

Murine breast cancer (4T1) cells were cultured in RPMI 1640 medium with 10% fetal bovine serum (FBS) and 1% penicillin-streptomycin and the cells were cultured at 37 ^o^C in a humidified atmosphere with 5% CO_2_.

### Cytoxicity assay

MTT assay was carried out to evaluate the toxicity of blank micelles and DOX-loaded micelles. Typically, 4T1 cells were seeded in 96-well plate at a density of 5000 per well. After incubation for 24 h, the culture medium was replaced with 200 μL fresh medium containing different concentrations of blank micelles and DOX-loaded micelles. After the cells were incubated for preselected time interval, the cells were subjected to MTT assay.

### Cellular imaging and tissue imaging

4T1 cells with a density of 1×10^5^ were seeded in glass dishes with a diameter of 35 mm. After incubation for 24 h, mPEATss micelles were added at a final TBIS of 30 μM, and the cells were incubated for another 1 h, 3 h and 5 h. In addition, lysosome and nucleus were dyed with lysotracker green and Hoechst 33342, respectively, to investigate the way that micelles entered the cells and the distribution of micelles in cells. Two-photon imaging was carried out on a Nikon ultrahigh resolution confocal laser microscope A1R MP+ with two-photon excitation to evaluate the two-photon imaging ability of micelles.

As for tissue imaging, BALB/c mice (n = 3) were injected with 200 μL mPEATss micelles (2 mg/mL) via tail vein. After 12 h, mice were sacrificed and the liver and the kidney were harvested and quickly washed with PBS for three times. Then the liver and the kidney were dyed with Hoechst 33342 for 30 min before being observed on a Nikon ultrahigh resolution two-photon confocal laser microscope A1R MP+.

### *Ex Vivo* and *in vivo* fluorescence imaging

mPEATss micelles (2 mg/mL) were injected via the tail vein of 4T1 tumor model bearing mice (n = 3). At selected time interval, mice were sacrificed and the major organs of heart, liver, spleen, lung and kidney as well as tumor were harvested. Afterwards, the organs and tumors were washed with PBS for several times, and the *ex vivo* fluorescence imaging was performed on a Maestro Imaging System. Tumors were further fixed with 4% formaldehyde, dehydrated and embed in paraffin to prepare tumor tissue sections for observation on CLSM with two-photon excitation.

As for *in vivo* fluorescence imaging, 4T1 tumor bearing BALB/c mice (n = 3) were injected with 200 μL mPEATss micelles (2 mg/mL) via tail vein. At preselected time intervals, mice were anesthetized by chloral hydrate, followed by being imaged with a Maestro Imaging System.

### *In vivo* pharmacokinetic study

To evaluate the DOX levels in plasma, BALB/c mice was injected with free DOX·HCl, DOX-loaded mPEAT micelles and DOX-loaded mPEATss micelles via tail vein at a dose of 5 mg DOX/kg body weight (three mice per group) at a volume of 200 μL (saline). At preselected times after the intravenous injection, the blood samples were collected by enucleation of the mice eyes and the mice were sacrificed immediately. The samples were processed according to previous work 32. The concentration of DOX was detected by HPLC (Agilent 1260, the mobile phase was composed by distilled water with 0.1% TFA and acetonitrile containing 0.1% TFA (70:30, v/v), 1 mL/min). The pharmacokinetic parameters were calculated via the pharmacokinetic software DAS 3.0 (Mathematical Pharmacology Professional Committee, People's Republic of China) by fitting to the two-compartment model.

### *In vivo* antitumor study

All animal experiments were carried out according to the institutional and NIH guidelines for the care and use of research animals. 4T1 cells at a density of 10^6^ were injected subcutaneously in the right back area of BALB/c mice (18 ± 2 g) to build subcutaneous 4T1 models. The mice were randomly divided into four groups (n = 7) when the tumor volume reached approximately 100 mm^3^ (tumor volume: V (mm^3^) = width × width × length, where width and length refer to the width and length of tumor). Afterwards, mice were treated with free DOX, DOX-loaded mPEATss micelles, DOX-loaded mPEAT micelles and saline, respectively, for four times in day 0, day 4, day 8 and day 12 at 5 mg DOX/kg body weight. Body weight of mice and volume changes were measured every two days to evaluate real-time side effects and antitumor efficacy. After a cycle of treatment (21 days), the mice were sacrificed and tumors and major organs were excised and fixed with 4% formaldehyde, which were treated with routine histopathological procedures and stained with hematoxylin and eosin (H&E) for histopathological evaluation. CD31, *K_i_*-67 and TUNEL were utilized to evaluate the angiogenesis, the apoptosis, and the cell proliferation of DOX-loaded micelles and free DOX 33-35.

## Results and Discussion

### Preparation and characterization of mPEATss and mPEAT

**Scheme 2:**
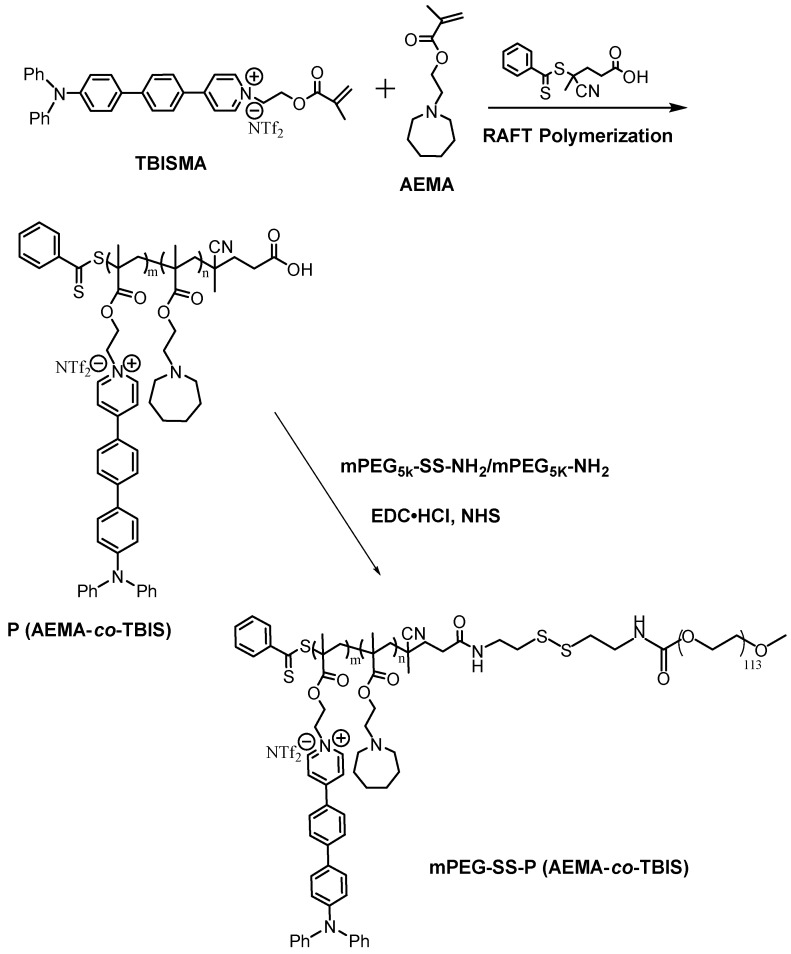
Synthetic route of mPEG-SS-P (AEMA-*co*-TBIS) and mPEG-P (AEMA-*co*-TBIS).

The synthetic route of copolymer was shown in Scheme 2. The newly designed fluorophore TBISMA was successfully prepared, which was confirmed by NMR in Figure S6 and Figure S7, and the AIE behavior of TBISMA was evaluated in mixed solution of DMSO and water with different water fraction (Figure S8), which exhibited great AIE feature and was expected to endow the copolymer with novel AIE characteristic. And a large two-photon absorption cross-section of 265 MG of TBIS further indicated that this fluorophore would be suitable for two-photon bioimaging. Poly (AEMA-*co*-TBIS) was synthesized via RAFT polymerization with the monomers of AEMA and TBISMA. The degree of polymerization (DP) of AEMA and TBIS were 8 and 1 based on the ^1^H NMR spectrum in Figure S9, respectively, and the molecular weight (Mn) of poly (AEMA-*co*-TBIS) was calculated as 2750. Meanwhile, the molecular weight distribution (MWD) of poly (AEMA-*co*-TBIS) was 1.17 determined by GPC with THF as the eluent (Figure S10). Afterwards, the hydroxyl group of mPEG-OH was activated with 4-nitrophenyl chloroformate (Figure S11B), and cystamine was conjugated to the terminal group of mPEG to obtain mPEG-SS-NH_2_ with an extent of cystamine conjugation about 94% (Figure S11C). Finally, mPEATss and mPEAT were obtained via amidation with mPEG-SS-NH_2_ and mPEG-NH_2_, respectively. According to the ^1^H NMR results (Figure S12 and Figure S13), the extent of amidation of mPEAT and mPEATss were both almost 100%, and the Mn of mPEAT and mPEATss were calculated as 7750 and 7900 based on the ^1^H NMR analysis, respectively. The PDI of mPEAT and mPEATss were further evaluated by GPC (Figure S11) in THF as 1.21 and 1.23, respectively.

### Characterization of DOX-loaded micelles and dual-responsive behavior

**Figure 1:**
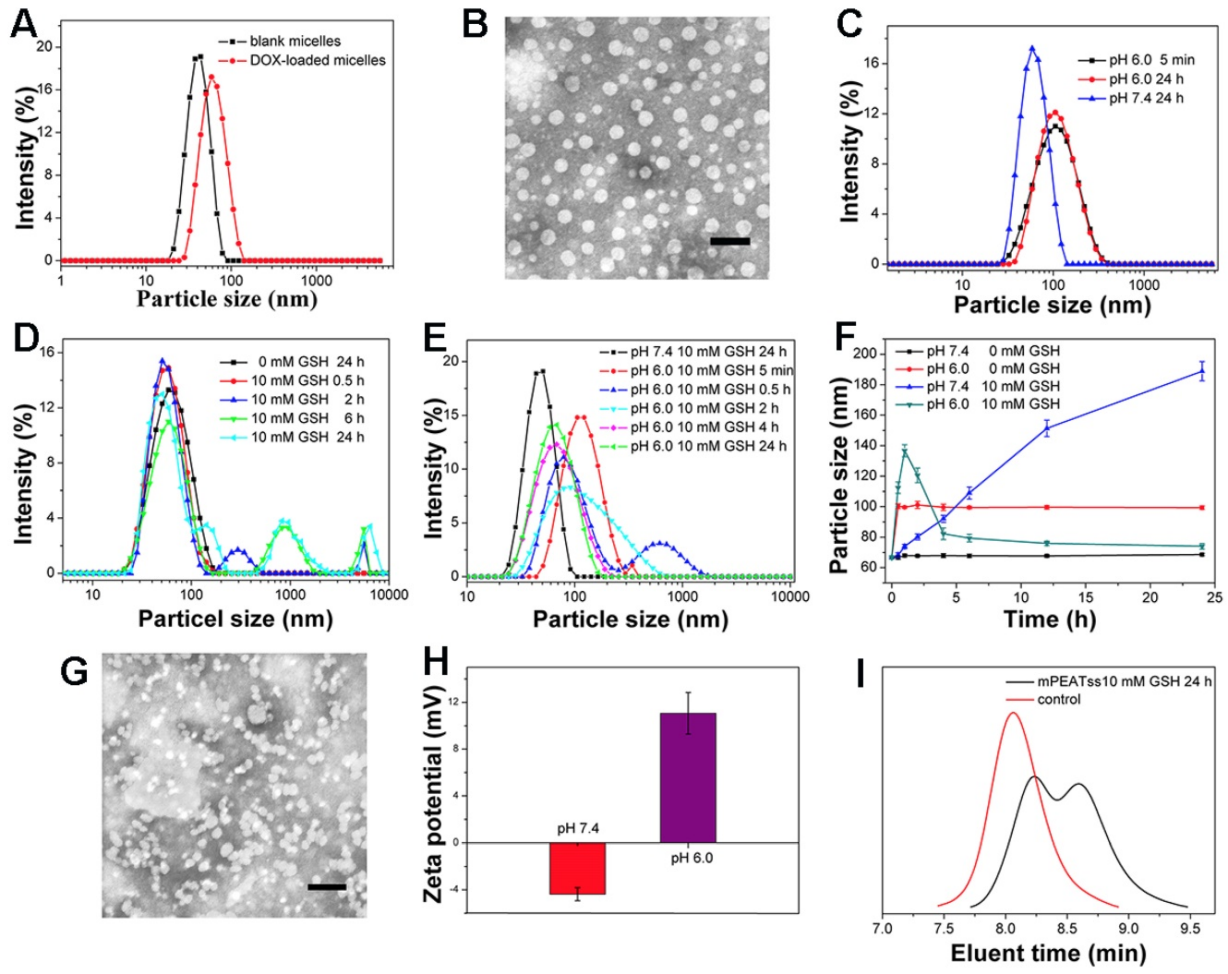
Characterization of mPEATss micelles. Blank and DOX-loaded mPEATss micelles (A). TEM image of DOX-loaded mPEATss micelles at pH 7.4 without GSH (B). Environment triggered disassembly of DOX-loaded mPEATss micelles at pH 6.0 (C), at medium contained 10 mM GSH (D) and at pH 6.0 with 10 mM GSH (E). Size changes of DOX-loaded mPEATss micelles monitored by DLS at medium contained GSH (0 or 10 mM) at pH 7.4 or pH 6.0 (F). TEM image of DOX-loaded mPEATss micelles after incubation at pH 6.0 with 10 mM GSH for 4 h (G). Zeta potential of DOX-loaded mPEATss micelles at pH 7.4 and pH 6.0 (H). GPC trace of mPEATss copolymer in THF after being incubating with 10 mM GSH for 24 h (I). Scale bar: 100 nm

Amphiphilic copolymer of mPEATss was expected to self-assemble into core-shell micellar structure in water. DLS results in Figure 1A indicated that the respective particle size of blank mPEATss micelles and DOX-loaded mPEATss micelles were 50.59 ± 0.62 nm (PDI: 0.16 ± 0.11) and 65.65 ± 0.83 nm (PDI: 0.10 ± 0.01), which would help to enhance the accumulation of drug-loaded nanocarriers at target sites via EPR effect 36. The decreased PDI of DOX-loaded micelles could be attributed to the hydrophobic interaction and π-π stacking between DOX and the hydrophobic Poly (AEMA-*co*-TBIS) segment. Meanwhile, blank and DOX-loaded mPEAT micelles exhibited similar particle size of 49.44 ± 0.91 nm (PDI: 0.18 ± 0.14) and 66.32 ± 1.07 nm (PDI: 0.15 ± 0.14) (Figure S14A), respectively. The morphology of DOX-loaded mPEATss micelles was further observed by TEM. As shown in Figure 1B, DOX-loaded mPEATss micelles possessed well-defined spherical morphology and the decreased particle size compared to that measured by DLS, which was attributed to the dehydration of micelles during sample preparation. Similar result was also found on mPEAT micelles in Figure S14B. Moreover, DOX-loaded mPEATss micelles could rapidly respond to acidic environment with increased particle size (Figure 1C), and GSH triggered disassembly of micelles could also be observed at medium contained 10 mM GSH (Figure 1D). More importantly, the disassembly of micelles would be much quicker in acidic medium with 10 mM GSH (Figure 1E). The environment triggered size changes of DOX-loaded micelles were monitored by DLS (Figure 1F), which fully confirmed that acidic environment and high level of GSH could synergistically promote the reassembly of micellar structure. The environment responsive behavior of mPEATss micelles was further evaluated by TEM, and obvious aggregates could be observed when micelles were incubated at pH 6.0 with 10 mM GSH for 4 h (Figure 1G), which might be due to the cleavage of disulfide bonds and newly formed micellar structure of Poly (AEMA-*co*-TBIS) at acidic medium. Furthermore, the charge-conversion of DOX-loaded micelles at acidic medium (Figure 1H) would potentially improve cellular uptake of DOX-loaded micelles. On the contrary, mPEAT micelles exhibited similar pH responsive behavior (Figure S14C) and similar charge-conversion behavior was observed (Figure S14H), but the micelles were stable at medium with 10 mM GSH (Figure S15D Figure S14E and Figure S14F). Moreover, TEM image in Figure S14G demonstrated the well formation of micellar structure of mPEAT micelles and there was no aggregates observed after incubation at pH 6.0 with 10 mM GSH for 4 h, which was in line with the result in Figure S14F. Moreover, the GSH-responsive ability of mPEATss was further evaluated by GPC via incubating micelles with 10 mM GSH for 24 h. The resulted samples were freeze-dried and evaluated by GPC in THF. As shown in Figure 1I, the PDI of mPEATss changed from unimodal to bimodal, suggesting the cleavage of the disulfide bond. In contrast, there was no obvious change in mPEAT copolymer (Figure S14I).

### AIE behavior study

**Figure 2:**
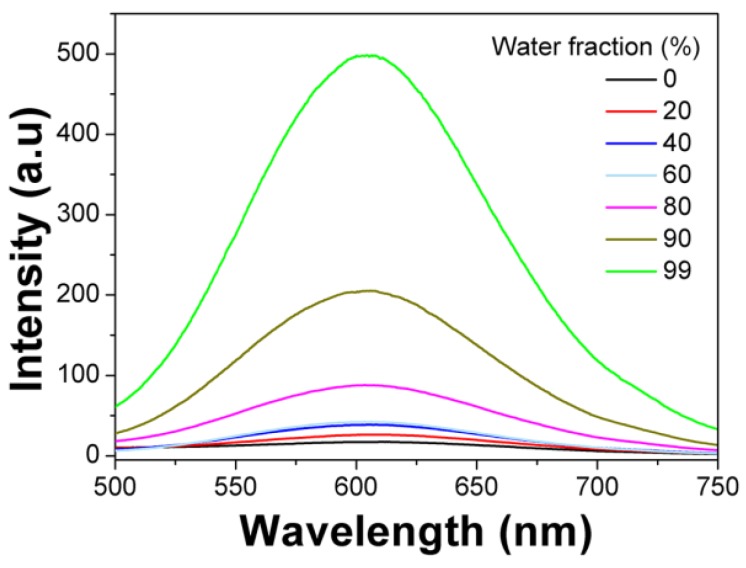
Emission spectra of mPEATss micelles in DMSO-water mixtures with different water fractions (λ_ex_ = 406 nm).

The AIE fluorophore TBIS endowed mPEATss micelles with great AIE feature, which emitted strong fluorescence in water due to that the TBIS group was in aggregation state in the hydrophobic core of micelle (Figure 2). Whereas, with the addition of DMSO, the TBIS group was gradually set free, resulting in the reduction of fluorescence (FL) intensity. The AIE quantum yields of TBIS, mPEAT and mPEATss copolymer were measured to be 16.28, 18.96 and 23.07, respectively, while the quantum yields of TBIS, mPEAT and mPEATss copolymer were all near to zero in dissolved state (in DMSO or THF). Moreover, the FL intensity of both mPEAT and mPEATss micelles was slightly reduced under acidic environment due to the PAEMA segment changed from hydrophobic to hydrophilic, resulting in looser micellar core and reduced aggregation state of TBIS. While high level of GSH did not show obvious influence on FL intensities owing to the GSH had no effect on micellar core (Figure S15A Figure S15B). The great AIE ability of the mPEATss micelle made it possible to apply in bioimaging.

### Biocompatibility and Two-photon excited fluorescence

**Figure 3:**
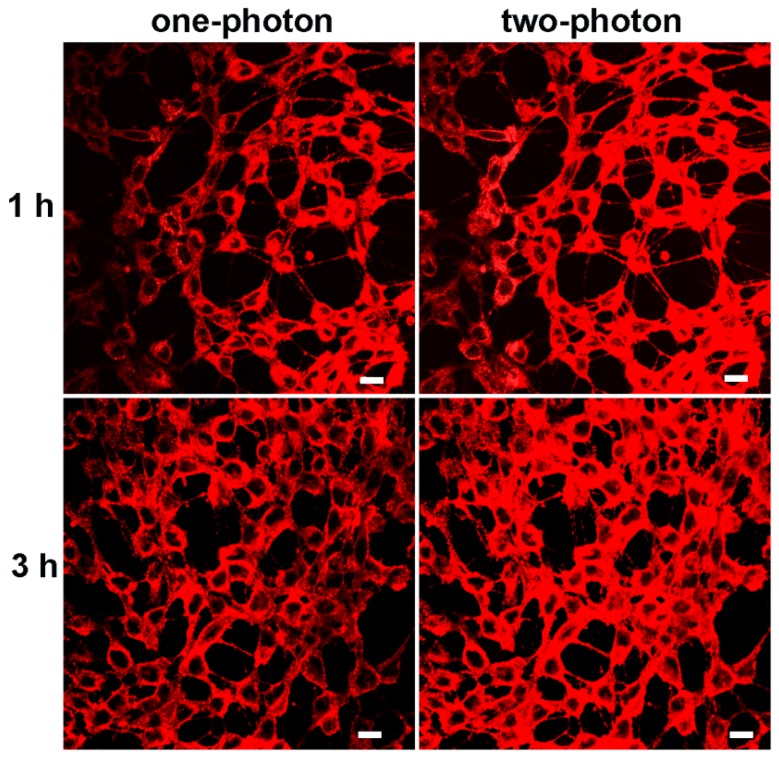
CLSM image of mPEATss micelles excited with 405 nm and 810 nm. Scale bar: 25 μm

Biocompatibility was the key issue for fluorescence probe to be used in bioimaging. The potential toxicities of mPEATss micelles and mPEAT micelles were studied by MTT assay against 4T1 cells (Figure S16A) and 3T3 cells (Figure S16B). Both of the two micelles exhibited great biocompatibility even the concentration of micelles reached to 400 μg/mL, which made these micelles available for biomedical application. Given the excellent AIE feature and great biocompatibility, CLSM with one-photon and two-photon excitation was further carried out to confirm the bioimaging ability of mPEATss micelles. As shown in Figure 3, strong red fluorescence in cytoplasm could be obviously observed in both one-photon and two-photon excitation, which became stronger over time. The inspiring two-photon imaging ability made these micelles suitable for serving as fluorescence probe for bioimaging. Moreover, Lysotracker Green and hoechst 33342 were further utilized to mark lysosomes and nucleus to investigate the endocytosis behavior and intracellular distribution of these fluorescent micelles (Figure S16), which further confirmed that these micelles were distributed in cytoplasm. Green fluorescence of lysosome and red fluorescence of micelles were well overlapped, suggesting the micelles were internalized by cells via endocytosis.

### *In vitro* drug release and cytotoxicity

**Figure 4:**
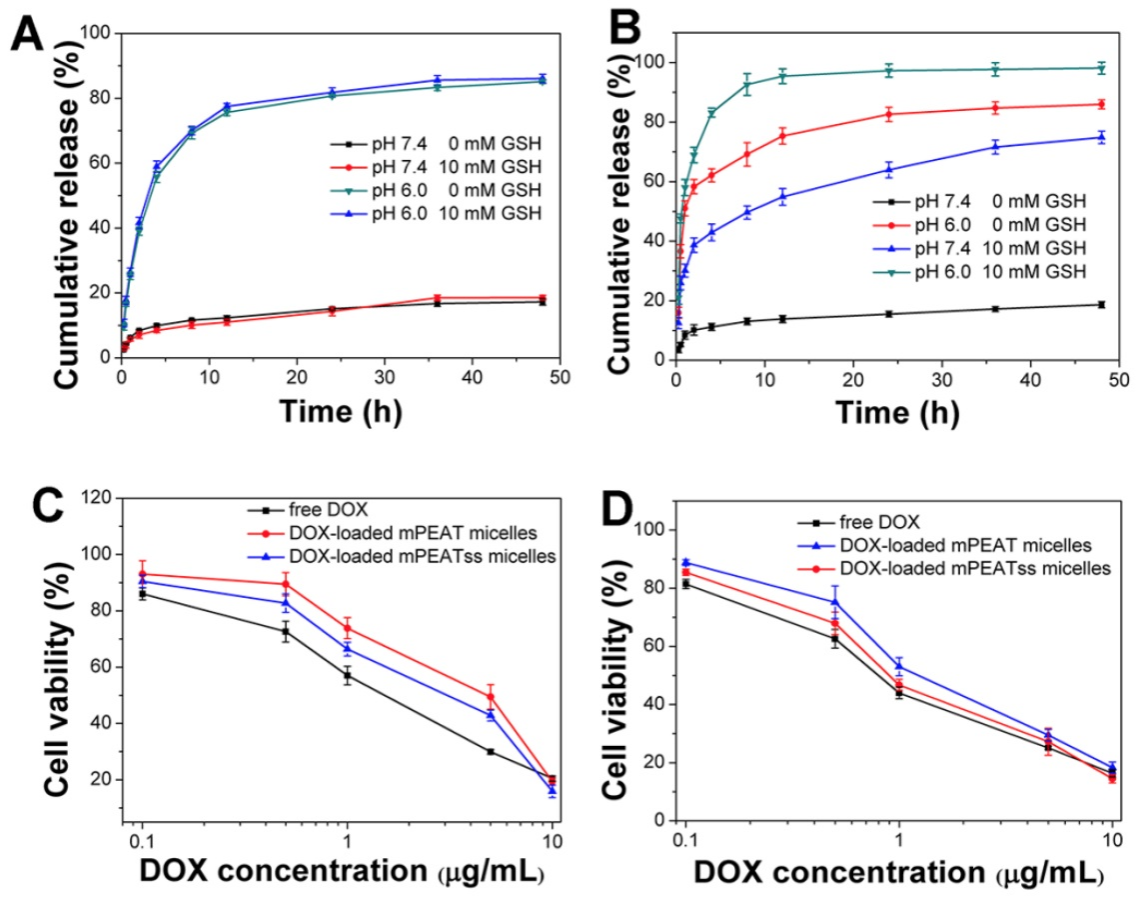
*In vitro* drug release of DOX-loaded micelles triggered with 10 mM GSH and acid: DOX-loaded mPEAT micelles (A) and DOX-loaded mPEATss micelles (B). Cytotoxicity of DOX-loaded mPEAT and mPEATss micelles against 4T1 cells for 48 h (C) and 72 h (D).

**Figure 5:**
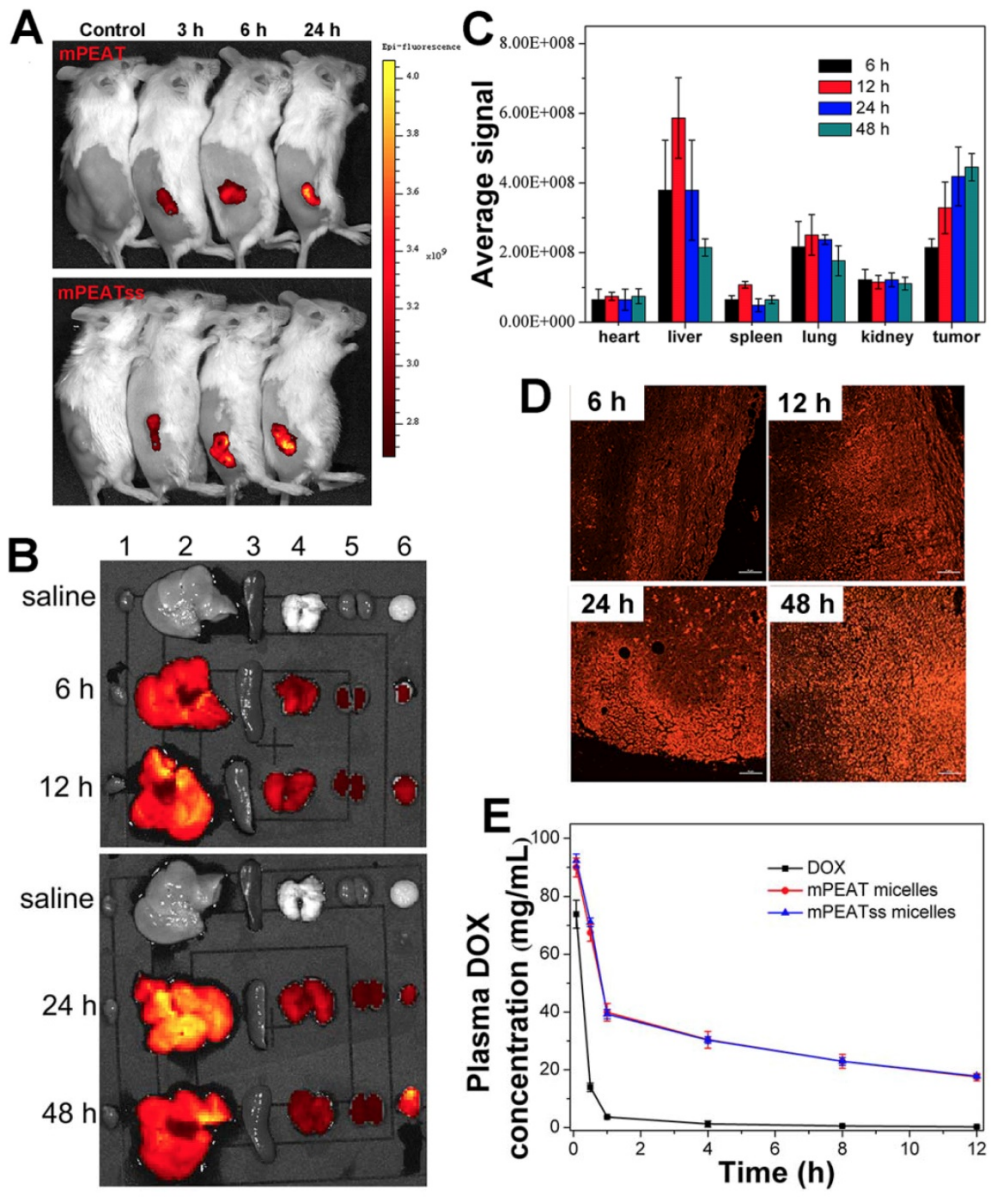
Fluorescent images of 4T1 tumor bearing BALB/c mice after treatment with mPEATss micelles at different monitoring times (A). *EX vivo* fluorescence imaging of major organs and tumors after treatment with mPEATss micelles for different time (B). Fluorescence signals of mPEATss micelles in major organs, counted by a Maestro imaging system (C) (n = 3). Two-photon images of tumor tissue sections excited at 810 nm (D) (Scale bar: 25 μm). Pharmacokinetic profiles after intravenous injection of DOX·HCl, DOX-loaded mPEAT micelles DOX-loaded mPEATss micelles in BALB/c mice (5 mg DOX per kg) (E).

In view of the great environment triggered disassembly of mPEATss micelles and mPEAT micelles, it would be desirable to trigger the drug release of DOX-loaded micelles by taking full advantage of the special environment of tumor. Drug release of DOX-loaded mPEAT micelles and mPEATss micelles were both accelerated by acid (Figure 4A and Figure 4B), but only DOX-loaded mPEATss micelles exhibited GSH triggered drug release, and the drug release of DOX-loaded mPEATss micelles was much quicker at pH 6.0 with the presence of 10 mM GSH. Therefore, mPEATss micelles would rapidly release the cargos at target sites and thus enhanced antitumor efficacy. Moreover, MTT assay was carried out to evaluate the tumor cells suppression ability of drug-loaded micelles. As shown in Figure 5C and Figure 5D, DOX-loaded mPEATss micelles exhibited better antitumor effect compared with DOX-loaded mPEAT micelles after incubation for 48 h and 72 h, respectively. The better antitumor efficacy of DOX-loaded mPEATss micelles might attribute to the pH and redox dual-triggered drug release. In addition, DOX-loaded mPEATss micelles showed similar antitumor efficacy with free DOX after incubation for 72 h, which was expected to enhance antitumor efficacy *in vivo*.

### *In vivo* and* ex vivo* imaging study

The distribution of nanocarriers *in vivo* was important for investigating the systemic toxicity of micelles. 4T1 tumor bearing mice was treated with blank mPEAT and mPEATss micelles at selected time interval, respectively. As shown in Figure 5A, obvious fluorescence signals could be observed both in mPEAT micelles and mPEATss micelles, which became stronger with time, indicating more micelles were accumulated in tumor sites. Moreover, mice (n = 3) treated with mPEATss micelles at preselected times were sacrificed, and major organs and tumors were isolated and washed with PBS for several times before imaging on a Maestro Imaging System. The results in Figure 5B demonstrated that the micelles were mainly metabolized via liver and mPEATss micelles could efficiently accumulate in tumor sites due to the EPR effect, which was expected to improve the bioavailability of antitumor drugs and minimize the side effects of drugs. The fluorescence in lung was attributed to the blood coagulation during anatomic process, which was hard to clean. Moreover, the semi quantitative of fluorescence intensity was counted by a Maestro Imaging System and the average signals were showed in Figure 5C, which was in accordance with the results in Figure 5a. Moreover, the tumors were made into sections to further evaluate the long-term accumulation of micelles via two-photon imaging. As shown in Figure 5C, under the same excitation intensity, the fluorescence signals of tumor sections increased with time, which further confirmed the effective accumulation of mPEATss micelles.

### *In vivo* pharmacokinetic study

It was essential for drug delivery system to minimize premature drug release during body circulation and maximize the circulation period, which was evaluated by *in vivo* pharmacokinetic study. As shown in Figure 5E and Table S1, DOX-loaded mPEAT micelles and mPEATss micelles exhibited significantly extended circulation time compared that with free DOX, which ensured that more drugs were delivered to the tumor sites. Moreover, compared with free DOX group parameters, half-life t_1/2_ (0.57 h), area under the curve (AUC, 35.31), the mPEAT micelles-encapsulated DOX and mPEATss micelles-encapsulated DOX exhibited obvious advantages: (i) enlarged t_1/2_ of 5.54 (9.72-fold) and 5.71 (10.02-fold), (ii) increased AUC of 344.17 (9.75-fold) and 352.04 (9.97-fold), respectively. In addition, the longer MRT and much slower clearance of micelles-encapsulated DOX further confirmed the DOX-loaded micelles would prolong circulation time *in vivo*. These results indicated that DOX-loaded micelles indeed obviously extended the blood circulation time and these micelles could be employed for *in vivo* DOX delivery.

### Tissue imaging study

**Figure 6:**
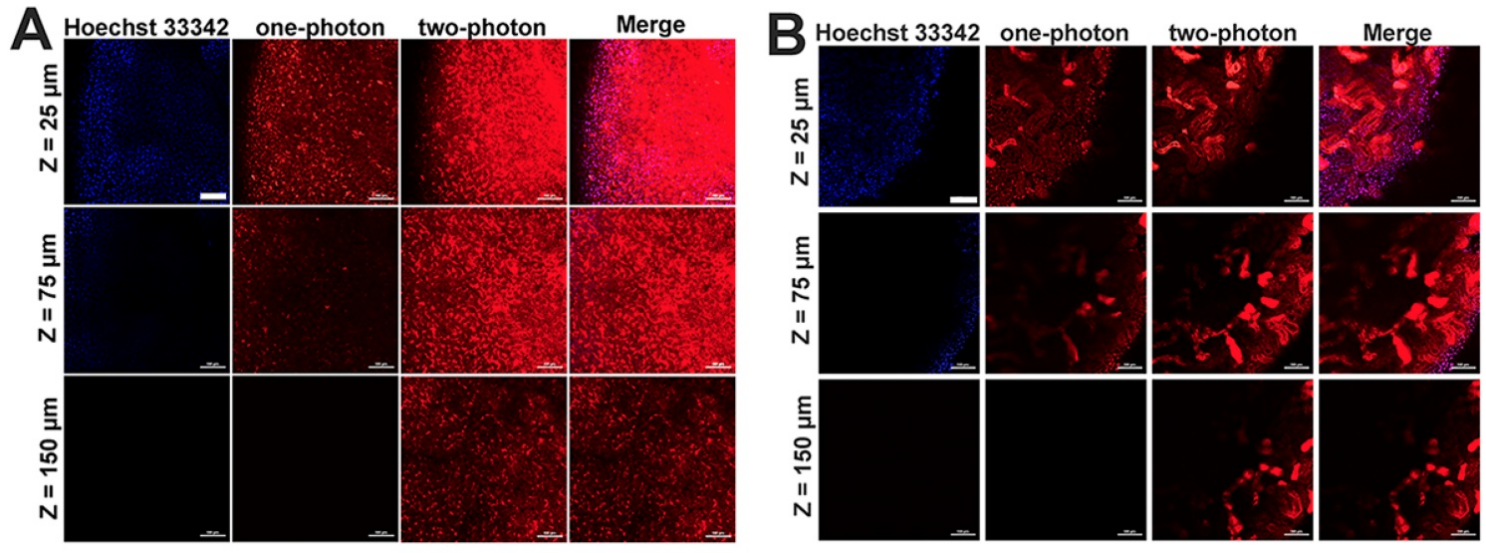
One-photon (excited at 405 nm) and two-photon (excited at 810 nm) images of hepatic tissue (A) and nephric tissue (B), where Merge referred to the overlap of Hoechst 33342 channel, one-photon channel and two-photon channel Scale bar: 100 μm

Given the two-photon cellular imaging ability was confirmed in Figure 3 and Figure 5C, tissue imaging was further carried out to evaluate the bioimaging ability of mPEATss micelles. After injection of blank mPEATss micelles for 12 h, the liver and the kidney were harvested and dyed with Hoechst 33342. As shown in Figure 6, red fluorescence signal of micelles and blue fluorescence signal of Hoechst 33342 could be found at the depth of 25 μm under one-photon excitation at 405 nm. However, the fluorescence signal of one-photon excitation reduced significantly with the increase of tissue depth, which might be due to the scattering of light. On the contrary, higher-quality images of both hepatic tissue (Figure 6A) and nephric (Figure 6B) tissue were obtained by two-photon excitation at 810 nm and the imaging depth could reach up to 150 μm, while the fluorescence signal excited by one-photon totally disappeared under the same condition. Thus, mPEATss micelles would be suitable for bioimaging owing to their two-photon NIR excitation, high penetration depth and high-quality images.

### *In vivo* antitumor study

**Figure 7:**
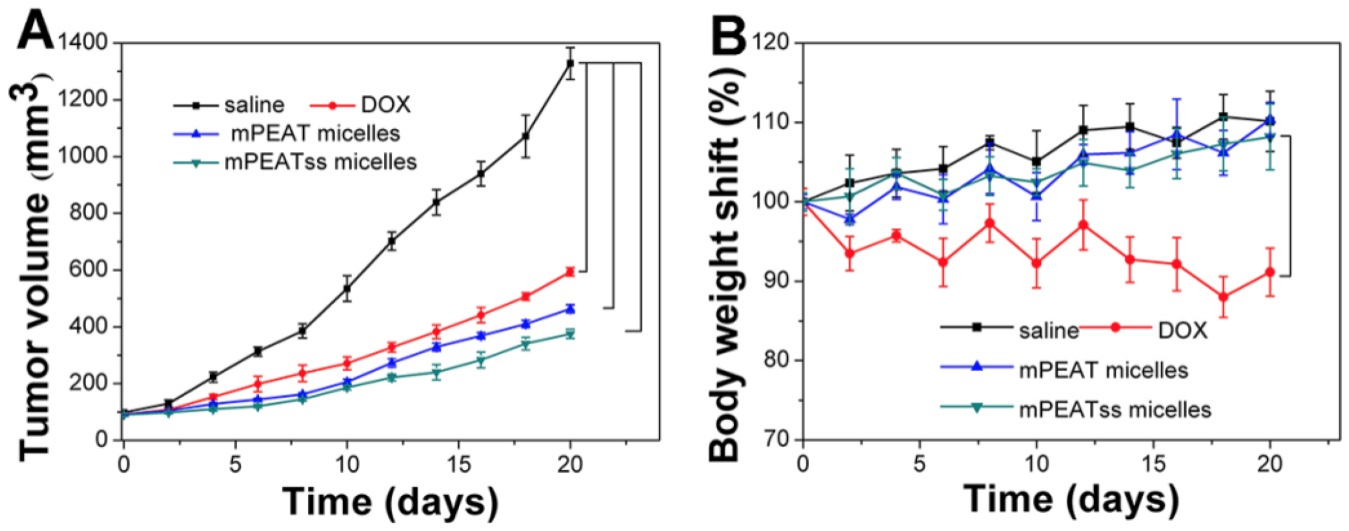
Volume of tumor treated with different formulations (A). body weight changes of mice treated with different formulations (B) (n = 6).

4T1 cancer bearing BALB/C mice were treated with saline, free DOX, DOX-loaded mPEAT micelles and DOX-loaded mPEATss micelles at a DOX dosage of 5 mg/kg body weight, respectively. Tumor volume was measured every two days to evaluate the antitumor efficacy in real-time and body weight changes were utilized to intuitively evaluate the side effects of different formulations. As shown in Figure 7A, saline treated mice did not show any tumor inhibition effect and the tumor volume increased rapidly, which reached about 1300 mm^3^ after a treatment cycle. However, free DOX, DOX-loaded mPEAT micelles and DOX-loaded mPEATss micelles exhibited great antitumor ability with tumor volume of ~590 mm^3^, ~460 mm^3^ and ~370 mm^3^, respectively. The better antitumor efficacy of DOX-loaded micelles was due to their long-circulating *in vivo* and significantly enhanced accumulation of drug. As expected, DOX-loaded mPEATss micelles showed the best tumor suppression ability and it hinted that their environment stimuli properties played an important role in the effective tumor inhibition. The clinical application of DOX was often limited by its severe side effects and obvious body weight loss could be observed in free DOX treated mice (Figure 7B). However, DOX-loaded mPEAT micelles and DOX-loaded mPEATss micelles treated mice did not show obvious body weight changes compared with that treated with saline, indicating that both of two micellar formulations were noninvasive and biocompatible. The enhanced antitumor efficacy and obviously reduced side effects made DOX-loaded mPEATss micelles great potential as a candidate for cancer therapy.

### Histological Studies and Immunohistochemical Analysis

The toxicity and antitumor efficacy of free DOX, DOX-loaded mPEAT micelles and DOX-loaded mPEATss micelles were further studied by histological studies of major organs and tumors after 21 days' treatment (Figure S17). Free DOX treated mice exhibited obvious cardiotoxicity, and obvious inflammation and focal necrosis could be easily found in lung and liver. However, there were no obvious abnormity in mice treated with DOX-loaded mPEAT micelles and DOX-loaded mPEATss micelles, which could be attributed to the intelligently controlled release of DOX* in vivo*. The significantly reduced side effect of DOX-loaded mPEATss micelles was expected to avoid DOX-associated toxicity and promote the long-term application of DOX. Meanwhile, DOX-loaded mPEATss micelles exhibited the most necrosis lesions in tumor than the other formulations, suggesting the enhanced antitumor efficacy. Therefore, it was proven that DOX-loaded mPEATss micelles could obviously reduce the side effects and improve the antitumor effect.

**Figure 8:**
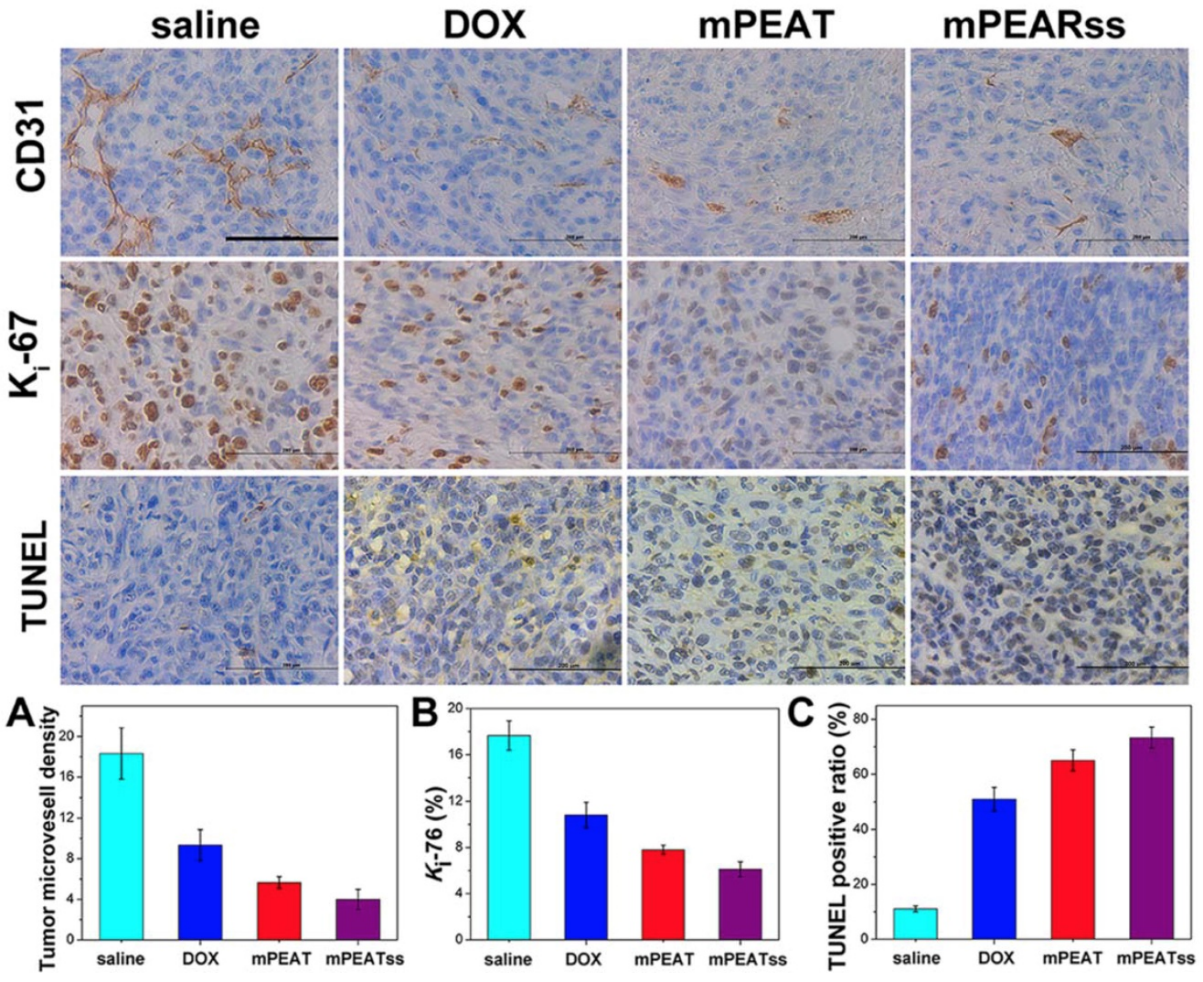
CD31, *K*_i_-67 and TUNEL immunohistochemical (IHC) analysis of 4T1 tumor (n=6, all tissue: 400×). The brown area in CD31, *K*_i_-67 and TUNEL images indicated positive. The capillary number was counted in each section to reflect MVD (p** < 0.01, DOX vs saline; p* < 0.05, DOX vs mPEAT; p** < 0.01, DOX vs mPEATss) (A); *K*_i_-67 density was calculated by *K*_i_-67-positive area to total area (p*** < 0.005, salne vs DOX; p* < 0.05, DOX vs mPEAT; p** < 0.01, DOX vs mPEATss) (B); apoptotic index were evaluated by counting the number of the apoptotic cells to the total cells in each microscopic field of view (p*** < 0.005, saline vs DOX; p** < 0.01, DOX vs mPEAT; p*** < 0.005, DOX vs mPEATss; p* < 0.05, mPEAT vs mPEATss) (C). Scale bars were 200 μm.

CD31 was utilized to mark tumor microvessel density (MVD) associating with tumor proliferation to evaluate antitumor efficacy and the lower MVD indicated the better antitumor efficacy. CD31 results in Figure 8A suggested that the tumors of mice treated with DOX-loaded mPEATss micelles exhibited the lowest proliferative capability, which could be attributed to the dual-responsive drug release. Meanwhile, *K*_i_-67, which was a key marker to reflect tumor proliferation analysis, was carried out to further evaluate tumor suppression ability. The results in Figure 8B showed that DOX-loaded mPEATss micelles exhibited the best tumor suppression ability compared with saline, free DOX and DOX-loaded mPEAT micelles, which was in accordance with Figure 8A. Moreover, TUNEL assay was also performed to evaluate tumor cell apoptosis. Only about 11% of apoptotic tumor cells were observed in saline treated mice. However, the apoptotic tumor cells of free DOX and DOX-loaded mPEAT and DOX-loaded mPEATss micelles were about 51%, 65% and 73% (Figure 8C), respectively. Therefore, the immunohistochemical analysis further indicated that DOX-loaded mPEATss micelles could effectively suppress tumor angiogenesis and tumor proliferation and cause apoptosis of tumor cells, which would improve antitumor efficacy.

## Conclusion

In this work, a multifunctional nanocarrier mPEATss micelle for cancer diagnosis and therapy has been developed based on a novel fluorophore TBIS with great AIE feature and two-photon imaging ability. mPEATss micelles exhibit great biocompatibility and excellent two-photon bioimaging performance. Utilizing the great tumor environment responsive properties of mPEATss micelles, drug release of DOX-loaded mPEATss micelles can be further accelerated with improved antitumor efficacy and less side effects *in vivo* compared with free DOX and DOX-loaded mPEAT micelles. Moreover, mPEATss micelles can efficiently accumulate in tumor sites, providing a potential candidate for cancer diagnosis and cancer therapy.

## Supplementary Material

Supplementary materials, figures, and table.

## Figures and Tables

**Scheme 1:**
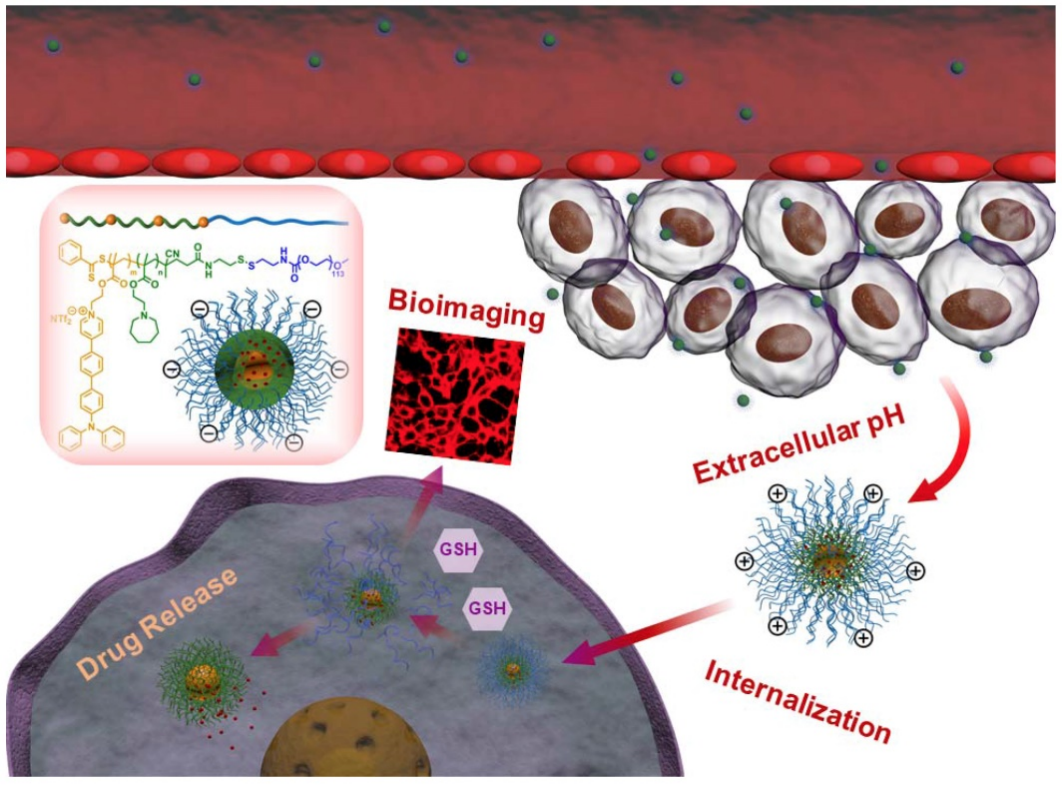
Illustration of DOX-loaded mPEATss micelles for pH-triggered charge-conversion enhanced cellular internalization, pH and redox dual triggered drug release and two-photon cell imaging.

## References

[1] Mei J, Leung NL, Kwok RT (2015). Aggregation-induced emission: together we shine, united we soar. Chem Rev.

[2] Kim D, Kang J, Wang T (2017). Two-photon in vivo imaging with porous silicon nanoparticles. Adv Mater.

[3] Tang Y, Kong X, Xu A (2016). Development of a two-photon fluorescent probe for imaging of nndogenous formaldehyde in living tissues. Angew Chem Int Ed Engl.

[4] Antaris AL, Chen H, Cheng K (2016). A small-molecule dye for NIR-II imaging. Nat Mater.

[5] Ebina T, Masamizu Y, Tanaka YR (2018). Two-photon imaging of neuronal activity in motor cortex of marmosets during upper-limb movement tasks. Nat Commun.

[6] Jiang M, Gu X, Lam J WY (2017). Two-photon AIE bio-probe with large Stokes shift for specific imaging of lipid droplets. Chem Sci.

[7] Zheng Z, Zhang T, Liu H (2018). Bright near-infrared aggregation-induced emission luminogens with strong two-photon absorption, excellent organelle specificity, and efficient photodynamic therapy potential. ACS Nano.

[8] Chen C, Liang Z, Zhou B (2018). In vivo near-infrared two-photon imaging of amyloid plaques in deep brain of alzheimer's disease mouse model. ACS Chem Neurosci.

[9] Feng Y, Zhu S, Antaris AL (2017). Live imaging of follicle stimulating hormone receptors in gonads and bones using near infrared II fluorophore. Chem Sci.

[10] Chen B, Feng G, He B (2016). Silole-based red fluorescent organic dots for bright two-photon fluorescence in vitro cell and in vivo blood vessel imaging. Small.

[11] Lei Z, Yue P, Wang X (2017). TPZ, a bright centrosymmetric two-photon scaffold for bioimaging. Chem Commun.

[12] Wang D, Lee MMS, Shan G (2018). Highly efficient photosensitizers with far-red/near-infrared aggregation-induced emission for in vitro and in vivo cancer theranostics. Adv Mater.

[13] Chang ZF, Jing LM, Chen B (2016). Rational design of asymmetric red fluorescent probes for live cell imaging with high AIE effects and large two-photon absorption cross sections using tunable terminal groups. Chem Sci.

[14] Qi J, Sun C, Li D (2018). Aggregation-induced emission luminogen with near-infrared-II excitation and near-infrared-I emission for ultradeep intravital two-photon microscopy. ACS Nano.

[15] Kataoka K, Harada A, Nagasaki Y (2012). Block copolymer micelles for drug delivery: Design, characterization and biological significance. Adv Drug Deliver Rev.

[16] Miyata K, Christie RJ, Kataoka K (2011). Polymeric micelles for nano-scale drug delivery. React Funct Polym.

[17] Peer D, Karp JM, Hong S (2007). Nanocarriers as an emerging platform for cancer therapy. Nat.

[18] Shi J, Kantoff PW, Wooster R (2017). Cancer nanomedicine: progress, challenges and opportunities. Nat Rev Cancer.

[19] Swierczewska M, Han HS, Kim K (2016). Polysaccharide-based nanoparticles for theranostic nanomedicine. Adv Drug Deliv Rev.

[20] Elsabahy M, Heo GS, Lim SM (2015). Polymeric nanostructures for imaging and therapy. Chem Rev.

[21] Ma B, Zhuang W Wang Y (2018). pH-sensitive doxorubicin-conjugated prodrug micelles with charge-conversion for cancer therapy. Acta Biomater.

[22] Guo X, Wei X, Jing Y (2015). Size changeable nanocarriers with nuclear targeting for effectively overcoming multidrug resistance in cancer therapy. Adv Mater.

[23] Wang J, Yang G, Guo X (2014). Redox-responsive polyanhydride micelles for cancer therapy. Biomaterials.

[24] Maiti C, Parida S, Kayal S (2018). Redox-responsive core-cross-linked block copolymer micelles for overcoming multidrug resistance in cancer cells. ACS Appl Mater Interfaces.

[25] Fleige E, Quadir MA, Haag R (2012). Stimuli-responsive polymeric nanocarriers for the controlled transport of active compounds: concepts and applications. Adv Drug Deliv Rev.

[26] Mura S, Nicolas J, Couvreur P (2013). Stimuli-responsive nanocarriers for drug delivery. Nat Mater.

[27] Hu J, Zhuang W, Ma B (2018). Redox-responsive biomimetic polymeric micelle for simultaneous anticancer drug delivery and aggregation-induced emission active imaging. Bioconjugate Chem.

[28] Li J, Meng X, Deng J (2018). Multifunctional micelles dually responsive to hypoxia and singlet oxygen: enhanced photodynamic therapy via interactively triggered photosensitizer delivery. ACS Appl Mater Interfaces.

[29] Su X, Ma B, Hu J (2018). Dual-responsive doxorubicin-conjugated polymeric micelles with aggregation-induced emission active bioimaging and charge conversion for cancer therapy. Bioconjugate Chem. 2018.

[30] Zhuang W, Xu Y, Li G (2018). Redox and pH dual-responsive polymeric micelles with aggregation-induced emission feature for cellular imaging and chemotherapy. ACS Appl Mater Interfaces.

[31] Alferiev IS, Connolly JM, Levy RJ (2005). A novel mercapto-bisphosphonate as an efficient anticalcification agent for bioprosthetic tissues. J Organomet Chem.

[32] Zhang Z, Zhang X, Xu X (2015). Virus-inspired mimics based on dendritic lipopeptides for eEfficient tumor-specific infection and systemic drug delivery. Adv. Funct. Mater.

[33] Mineo TC (2004). Prognostic impact of VEGF, CD31, CD34, and CD105 expression and tumour vessel invasion after radical surgery for IB-IIA non-small cell lung cancer. J Clin Pathol.

[34] Burcombe RJ, Makris A, Richman PI (2005). Evaluation of ER, PgR, HER-2 and Ki-67 as predictors of response to neoadjuvant anthracycline chemotherapy for operable breast cancer. Br J Cancer.

[35] Labat-Moleur F, Guillermet C, Lorimier P (1998). TUNEL apoptotic cell detection in tissue sections: critical evaluation and improvement. J Histochem Cytochem.

[36] Bae Y, Kataoka K (2009). Intelligent polymeric micelles from functional poly (ethylene glycol)-poly (amino acid) block copolymers. Adv Drug Deliv Rev.

