# Supplementary material for: Two-photon AIE luminogen labeled multifunctional polymeric micelles for theranostics
Source: Theranostics. 2019 Aug 27;9(22):6618–30. doi: 10.7150/thno.33901 (PMC6771243; doi:10.7150/thno.33901)
Supplement: Supplementary file 1 — Supplementary materials, figures, and table. [file thnov09p6618s1.pdf]

Supporting Information

Two-Photon *AIE Luminogens* labeled Multifunctional Polymeric Micelles for Theranostics

Weihua Zhuang[1], Boxuan Ma[1], Jun Hu, Jizhou Jiang, Gaocan Li, Li Yang* and Yunbing Wang*

National Engineering Research Center for Biomaterials, Sichuan University, 29 Wangjiang Road, Chengdu 610064, China. E-mail: yanglisc@scu.edu.cn (Dr. Li Yang); yunbing.wang@scu.edu.cn (Prof. Yunbing Wang, Director of National Engineering Research Center for Biomaterials)

[1]These authors contribute equally to this work.

Materials

1-Bromo-4-iodobenzene, 4-pyridylboronic acid, tetrakis (triphenylphosphine) palladium (0) (Pd $(PPh_3)_4$), 4-(diphenylamino) phenylboronic acid, 2-Iodoethanol, lithium bis (trifluoromethanesulphonyl) imide and methacryloyl chloride were obtained from Adamas Reagent, Ltd (Shanghai, China). 1-Ethyl-3-(3-dimethylaminopropyl) carbodiimide hydrochloride (EDC • HCl) and N-Hydroxysuccinimid (NHS), 4-Cyanopentanoic acid dithiobenzoate and 3-(4, 5-Dimethyl-thiazol-2-yl)-2, 5-diphenyl tetrazolium bromide (MTT) were obtained from Sigma-Aldrich and used as received.

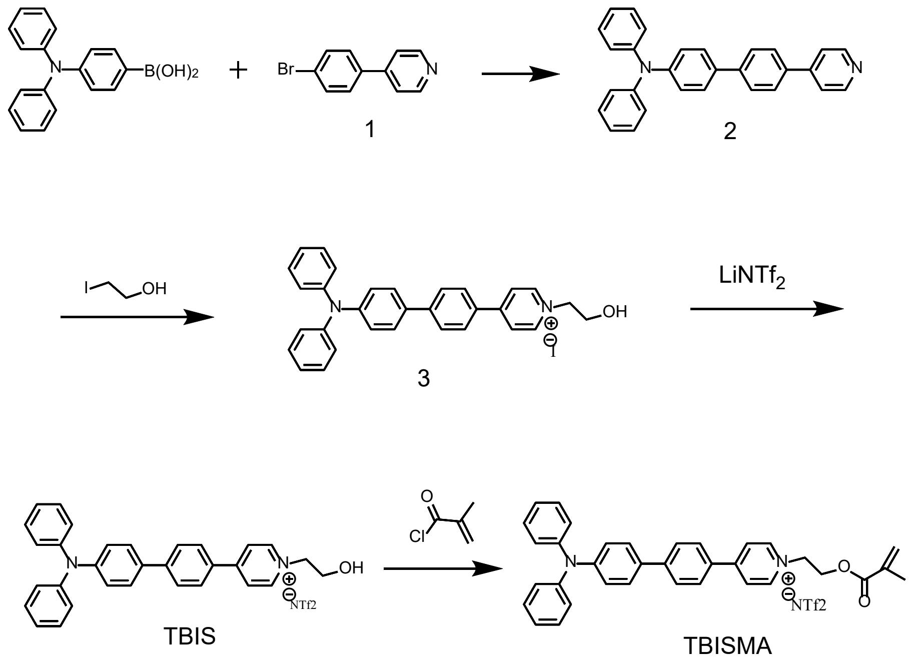

Figure S1. Synthetic route of TBISMA

Synthesis of TBISMA

4-(4-Bromophenyl) pyridine (compound 1) was synthesized according to previous work[1]. Compound 1 (2.00 g, 8.54 mmol), Pd $(PPh_3)_4$ (448.75 mg, 0.39 mmol), sodium carbonate (2.72 g, 25.63 mmol) and 4-(diphenylamino) phenylboronic acid (2.59 g, 8.97 mmol) were added to a 250 mL three-necked round-bottomed flask with condenser pipe charged. After being vacuumized and inflated with argon (Ar) for three times, degassed mixed solution of 100 mL toluene, 30 mL ethyl alcohol (EtOH) and 10 mL water (H_2O) were added under Ar atmosphere. The reaction was performed at 110 ^{o}C for 30 h. The resulted solution was filtered and the solvent was removed under reduced pressure. The crude products were purified by flash chromatography with a mixture solution of ethyl acetate (EtOAc) and petroleum ether (PE) to obtain compound 2 (1:3, V/V) (yield 3.18 g, 93.40%). ^{1}H NMR (DMSO-d_6,

400 MHz): δ = 8.67-8.72 (d, 2H), 7.68-7.76 (m, 4H), 7.60-7.65 (d, 2H), 7.51-7.56 (d, 2H), 7.25-7.33 (m, 8H), 7.13-7.19 (m, 6H), 7.04-7.10 (t, 2H) ppm.

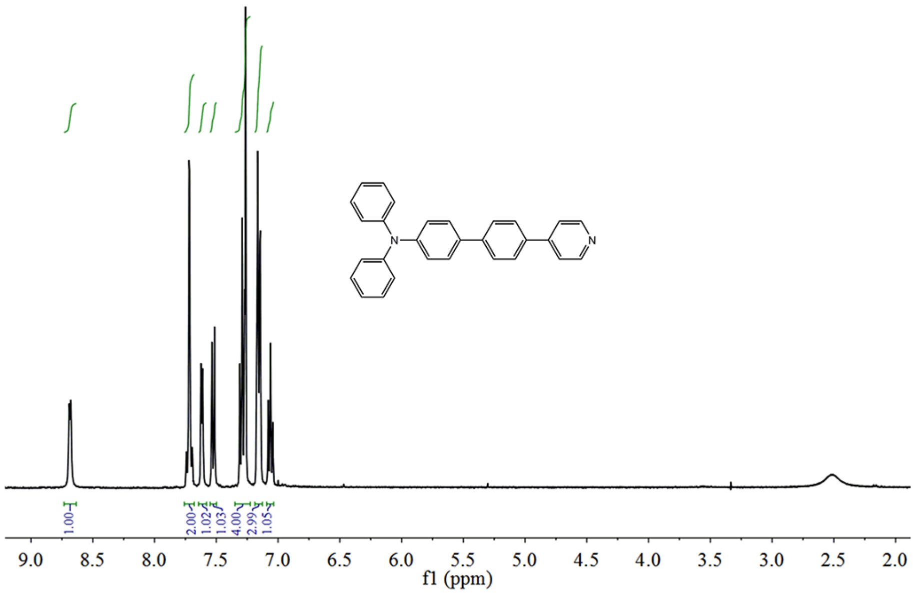

Figure S2. ^{1}H NMR spectrum of compound 2 in DMSO-d_6.

Compound 2 (2.00 g, 5.02 mmol) and 2-Iodoethanol (4.32 g, 25.09 mmol) were dissolved in 50 mL THF and the solution was stirred at 80 °C for 48 h. The resulted solution was concentrated under reduced pressure and the crude products were purified via flash chromatography with a mixed solution of DCM and MeOH (10:1, V/V) to obtain compound 3 (yield 2.41 g, 84.18%). ^{1}H NMR (DMSO-d_6, 400 MHz): δ = 8.97-9.04 (d, 2H), 8.54-8.61 (d, 2H), 8.14-8.22 (d, 2H), 7.88-7.97 (d, 2H), 7.71-7.79 (d, 2H), 7.31-7.40 (m, 4H), 7.02-7.15 (m, 8H), 4.59-4.66 (t, 2H), 3.85-3.92 (t, 2H) ppm.

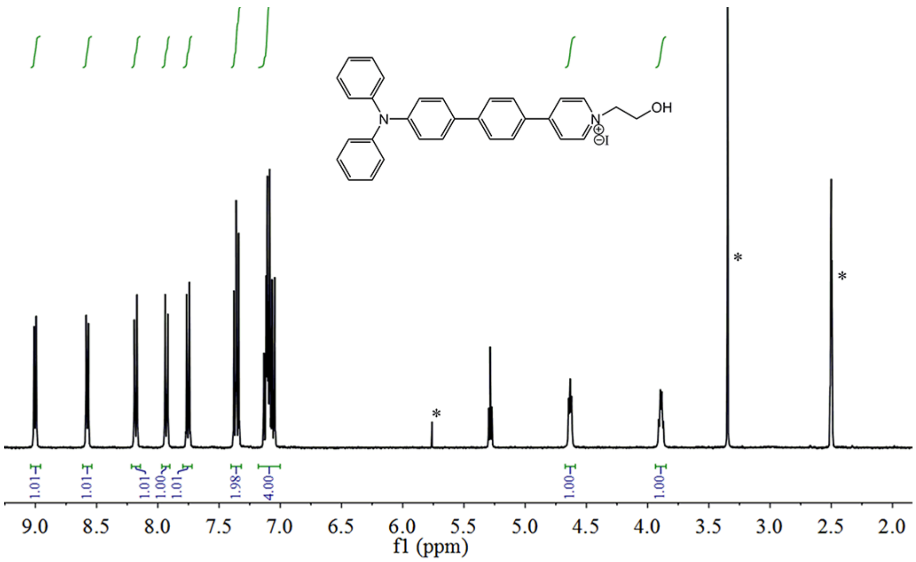

Figure S3. ^{1}H NMR spectrum of compound 3 in DMSO-d_6.

Compound 3 (1.5 g, 2.63 mmol) was dissolved in a mixed solution of 40 mL DCM and 10 mL H_2O and bis (trifluoromethanesulphonyl) imide (1.51 g, 5.26 mmol) was added. After stir at room temperature for 24 h, 200 mL DCM was added and the resulted solution was extracted with brine and the organic layer was dried over anhydrous $NaSO_4$. TBIS was obtained by flash chromatography with a mixture solution of DCM and MeOH (10:1, V/V) (yield 1.85 g, 97.22%). ^{1}H NMR (DMSO-d_6, 400 MHz): δ = 8.97-9.04 (d, 2H), 8.54-8.61 (d, 2H), 8.14-8.22 (d, 2H), 7.88-7.97 (d, 2H), 7.71-7.79 (d, 2H), 7.31-7.40 (m, 4H), 7.02-7.15 (m, 8H), 4.59-4.66 (t, 2H), 3.85-3.92 (t, 2H) ppm. ^{19}F (DMSO-d_6, 376 MHz): δ = -78.79 ppm.

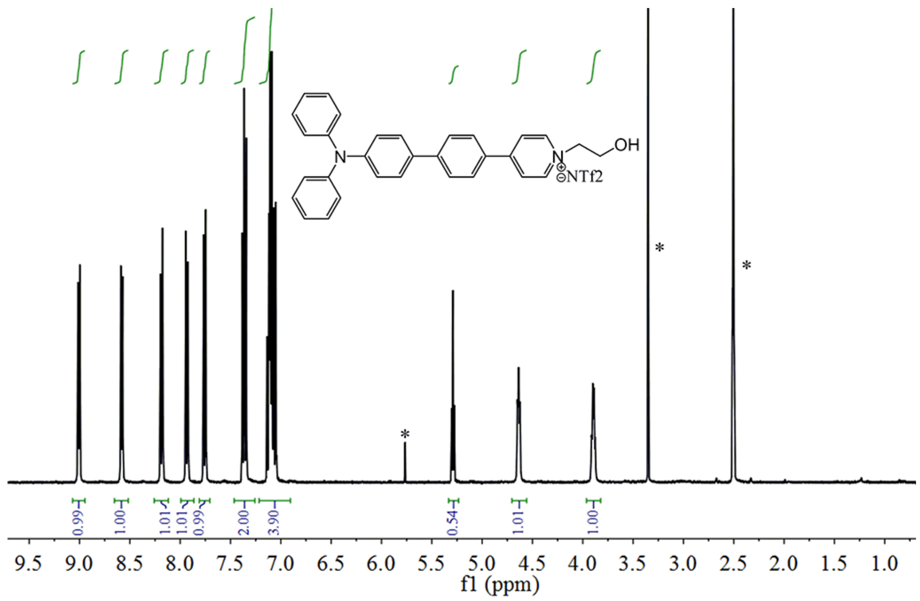

Figure S4. ^{1}H NMR spectrum of TBIS in DMSO-d_6.

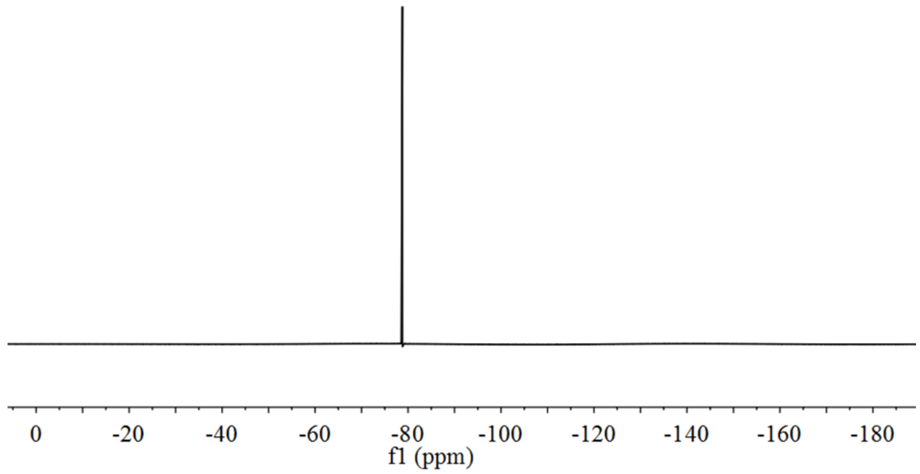

Figure S5. ^{19}F NMR spectrum of TBIS in DMSO-d_6 .

Under Ar atmosphere, TBIS (1.00 g, 1.38 mmol) and triethylamine (0.42 g, 4.15 mmol) were dissolved in 50 mL dry DCM. Then methacryloyl chloride (0.22 g, 2.07 mmol) dissolved in 15 mL dry DCM was dropwise added in ice bath. The mixed solution was stirred at room temperature for 24 h and monitored by TLC to make sure TBIS was totally exhausted. The resulted solution was washed with $NaHCO_3$ and

brine for four times, respectively. The organic layer was dried over anhydrous $NaSO_4$ and TBISMA was obtained via recrystallized from DCM and PE. ^{1}H NMR (DMSO-d_6, 400 MHz): δ =9.10-9.16 (d, 2H), 8.59-8.67 (d, 2H), 8.18-8.24 (d, 2H), 7.91-7.99 (d, 2H), 7.73-7.80 (d, 2H), 7.32-7.41 (m, 4H), 7.03-7.17 (m, 8H), 6.03-6.07 (s, 1H), 5.71-5.74 (s, 1H), 4.92-4.98 (t, 2H), 4.62-4.68 (t, 2H) ppm

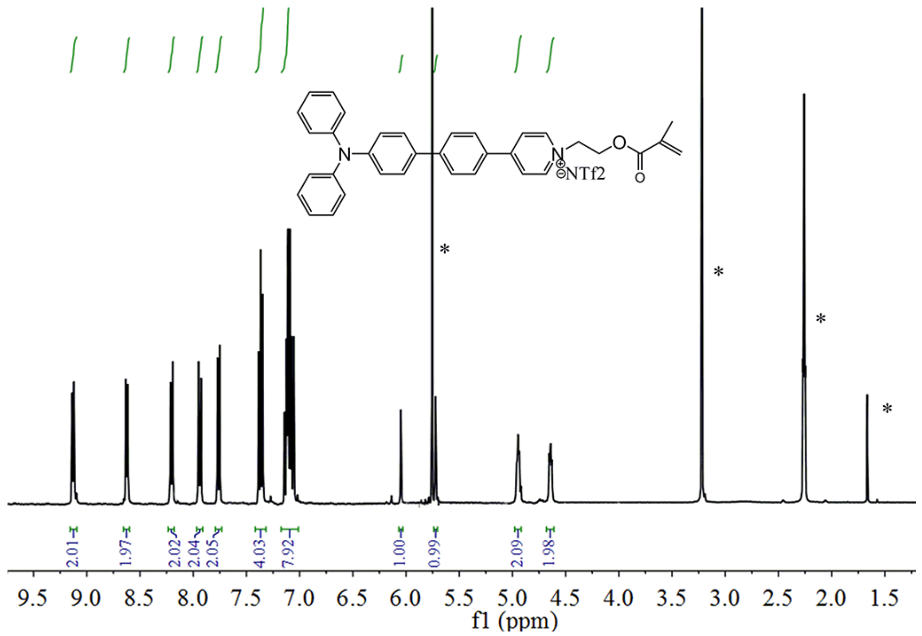

Figure S6. ^{1}H NMR spectrum of TBISMA in DMSO-d_6.

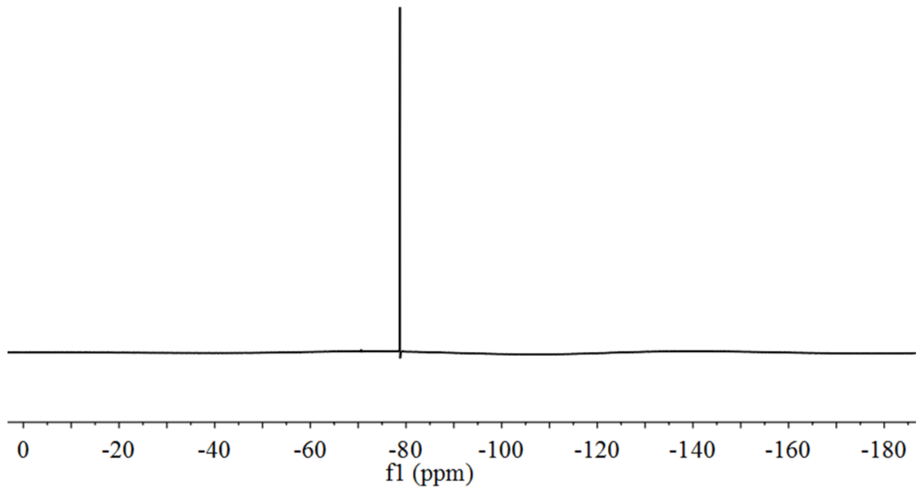

Figure S7. ^{19}F NMR spectrum of TBISMA in DMSO-d_6.

The measurement of two-photon absorption cross-section

The two-photon absorption cross-section was calculated based on the following formula[2]: $\boldsymbol{\sigma_2 = \sigma_1 (F_2 / F_1)(c_1 / c_2)(\phi_1 / \phi_2)}$ from the reported literatures (J. Phys. Chem. C 2015, 119, 27630−27638;). Rhodamine B was used as standard whose two-photon absorption cross-section could be found from www.drbio.cornell.edu/cross_sections.htm. Subscript 2 referred to the sample and subscript 1 referred to Rhodamine B. $\boldsymbol{\sigma_1}$ and $\boldsymbol{\sigma_2}$ represented two-photon absorption cross-sections of the sample and Rhodamine B, respectively. ***F*** stands for the integrated intensity of fluorescence spectra after being corrected with the instrumental spectral response function. ***c*** refers to the molar concentration of fluorophores in the sample solutions. $\boldsymbol{\phi}$ is one-photon excited fluorescence quantum efficiency. We have added this part in the Supporting Information.

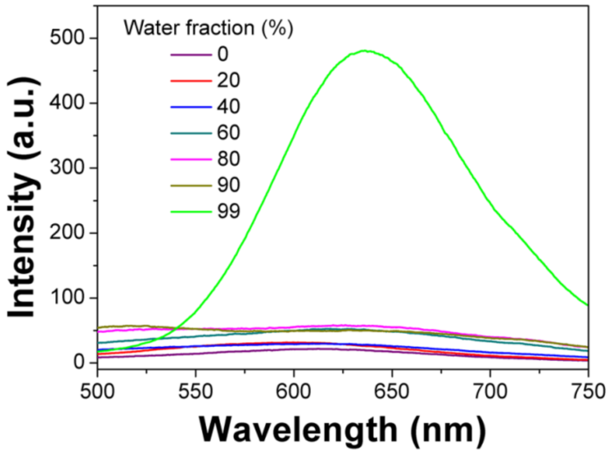

Figure S8. Emission spectra of TBISMA in DMSO-water mixtures with different water fractions

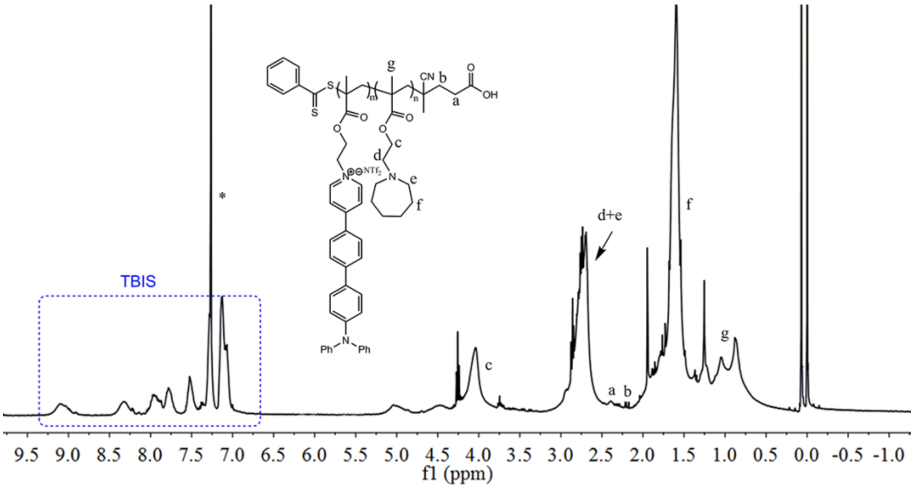

Figure S9. ^{1}H NMR spectrum of Poly (AEMA-*co*-TBIS) in $CDCl_3$.

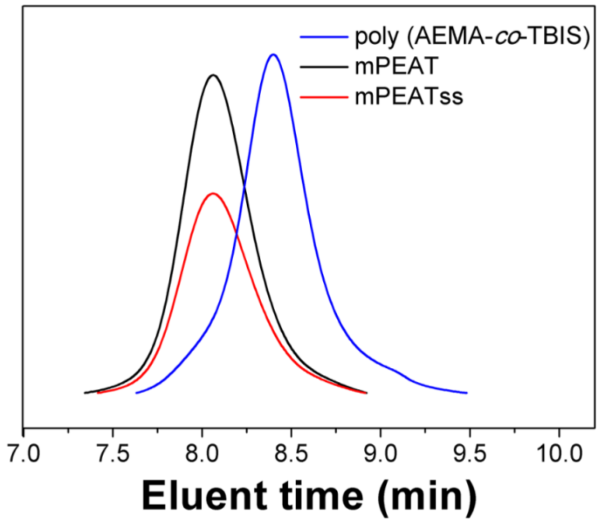

Figure S10. GPC traces of Poly (AEMA-*co*-TBIS), mPEAT and mPEATss in THF.

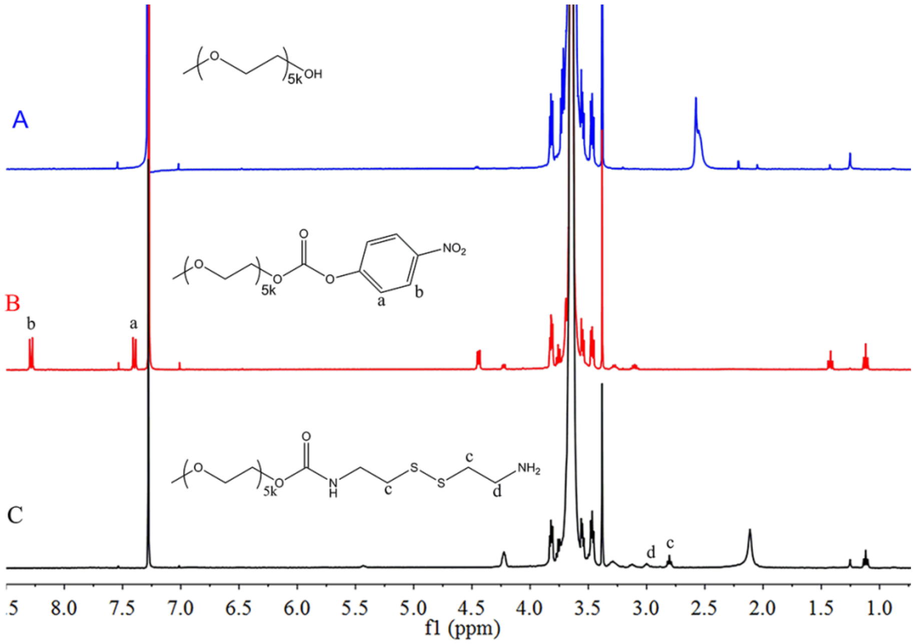

Figure S11. ^{1}H NMR of $mPEG_{5k}$-OH in $CDCl_3$ (A). ^{1}H NMR of $mPEG_{5k}$-NO_2 in

$CDCl_3$ (B). ^1H NMR of $mPEG_{5k}$-SS-NH_2 in $CDCl_3$ (C).

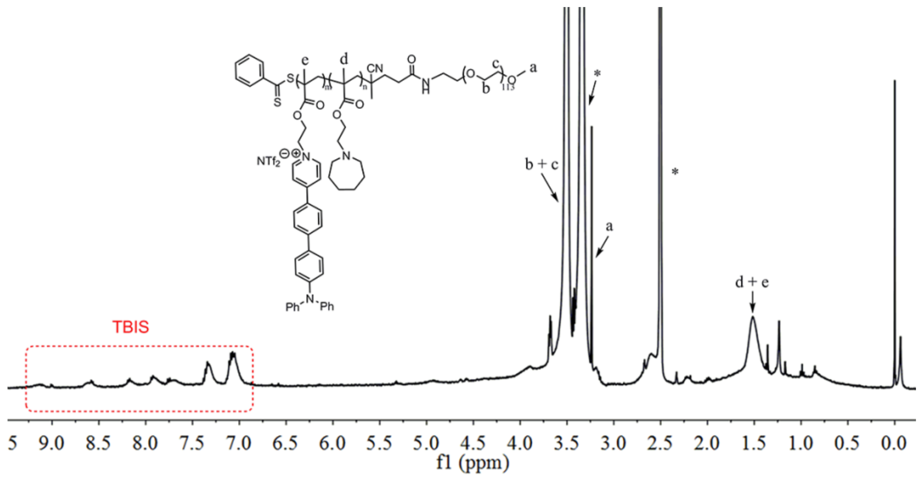

Figure S12. ^1H NMR of mPEG-Poly (AEMA-*co*-TBIS) in DMSO-d_6.

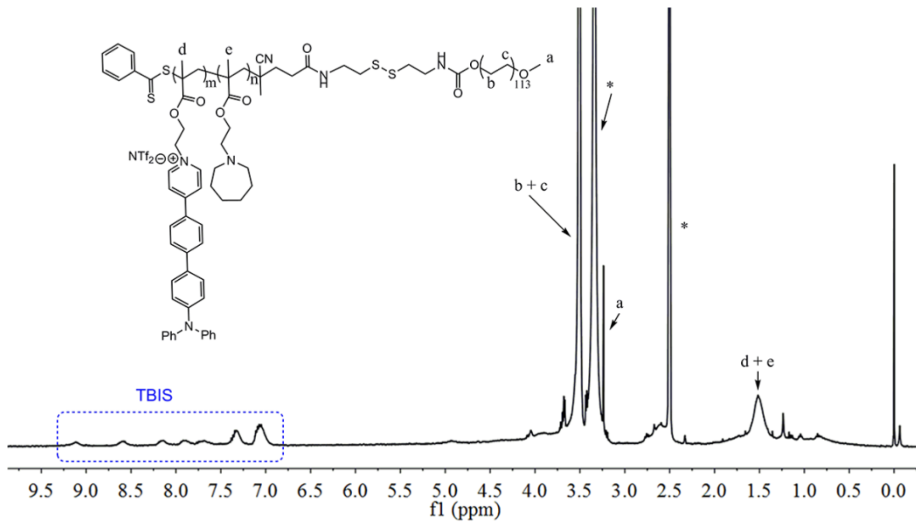

Figure S13. ^1H NMR of mPEG-SS-Poly (AEMA-*co*-TBIS) in DMSO-d_6.

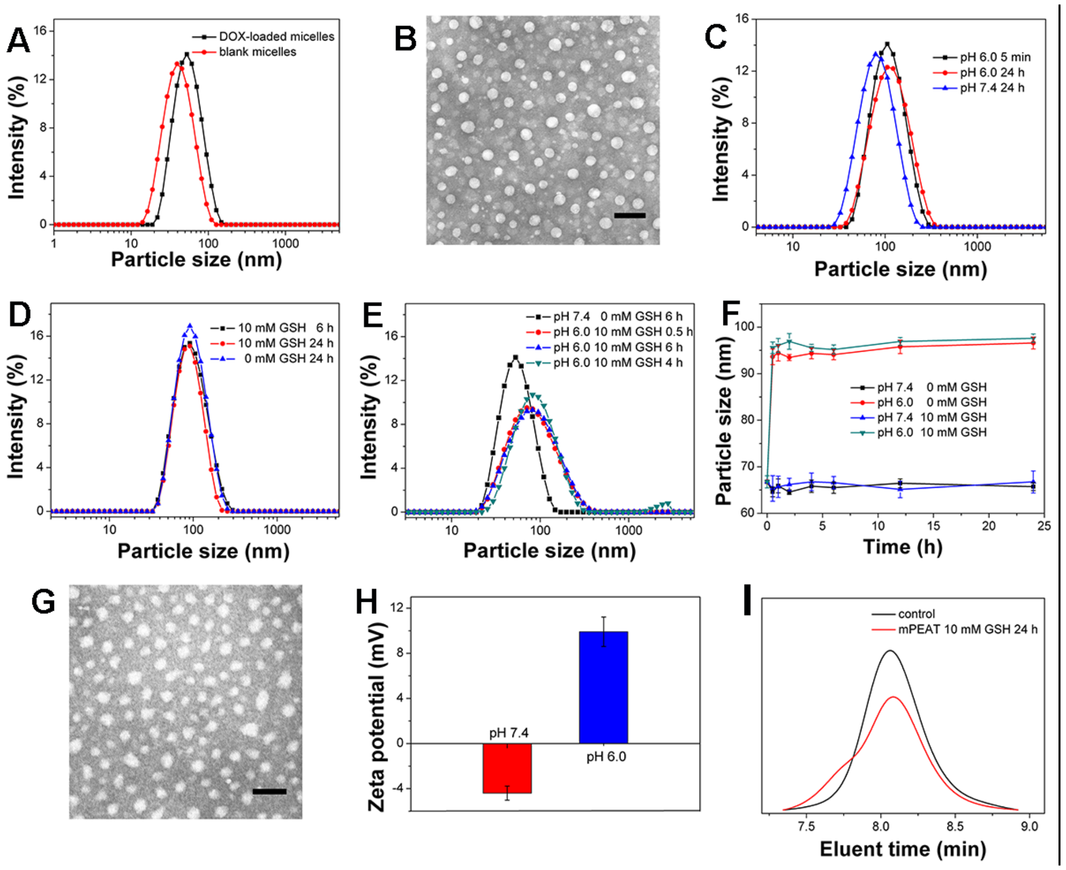

Figure S14. Characterization of mPEAT micelles. Blank and DOX-loaded mPEAT micelles (A). Environment triggered disassembly of DOX-loaded mPEAT micelles at pH 6.0 (B), at medium contained 10 mM GSH (C) and at pH 6.0 with 10 mM GSH (D). Size changes of DOX-loaded mPEAT micelles monitored by DLS at medium contained GSH (0 or 10 mM) at pH 7.4 or pH 6.0 (E). Zeta potential of DOX-loaded mPEAT micelles at pH 7.4 and pH 6.0 (F). TEM image of DOX-loaded mPEAT micelles at pH 7.4 without GSH (G) and that incubated at pH 6.0 with 10 mM GSH for 4 h (H). GPC trace of mPEAT copolymer in THF after being incubating with 10 mM GSH for 24 h (I). Scale bar: 100 nm.

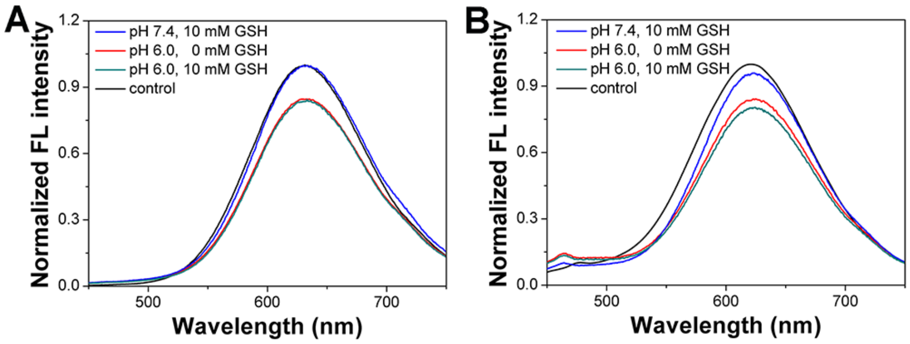

Figure S15. Emission spectra of mPEATss micelles (A) and mPEAT micelles (B) in different mediums.

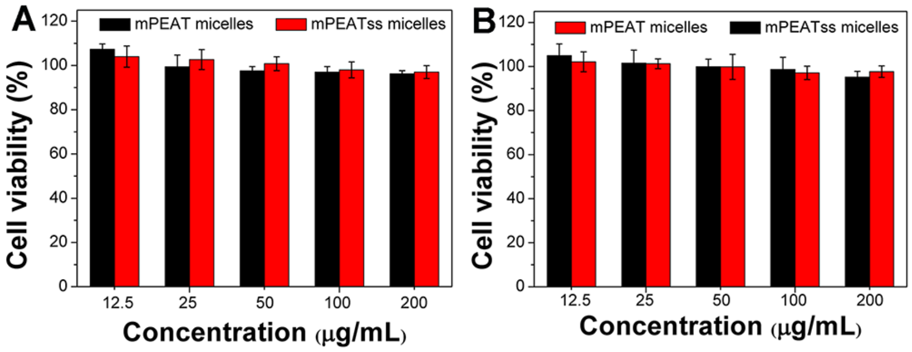

Figure S16. Cytotoxicity of blank mPEATss micelles and blank mPEAT micelles against 4T1 cells (A) and 3T3 cells (B).

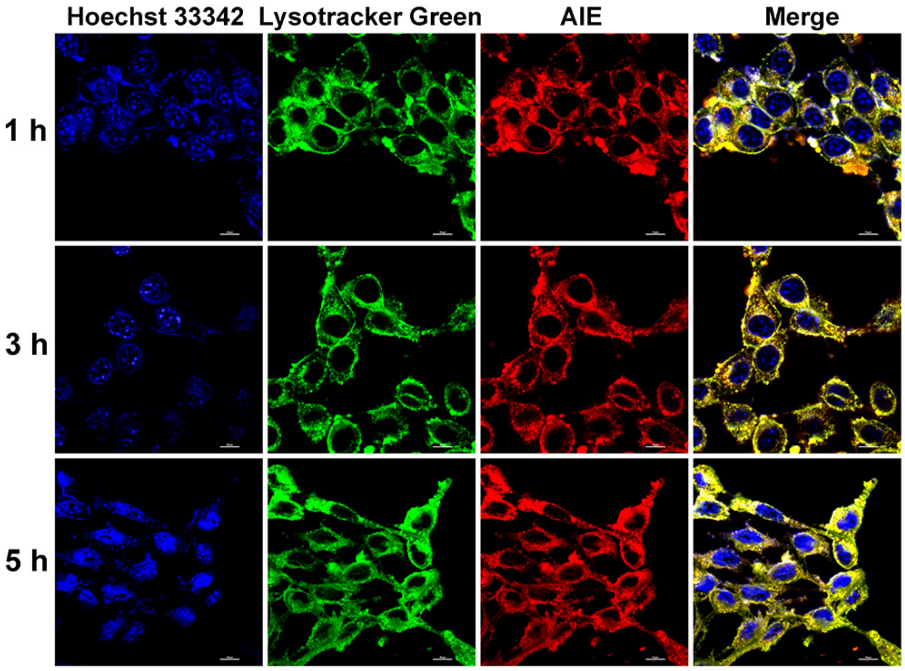

Figure S17. CLSM images 4T1 cells after incubating with blank mPEATss micelles

(0.4 mg/mL) with the final concentration of TBP-ET of 50 μM. Lysosomes were stained with Lysotracker (green) and nucleus was stained with Hoechst 33342 (blue).

Table S1. Pharmacokinetic parameters of DOX after intravenous injection of DOX·HCl, DOX-loaded mPEAT micelles DOX-loaded mPEATss micelles in BALB/c mice (5 mg DOX per kg) (F) (mean ± SD, n = 3).

Parameters*	DOX	mPEAT micelles	mPEATss micelles
AUC (μg h/mL)	35.31	344.17	352.04
$t_{1/2}$ (h)	0.57	5.54	5.71
MRT (h)	0.39	3.12	3.09
CL (L/h/kg)	0.23	0.047	0.048

*AUC: area under the curve, $t_{1/2}$: half-life time, MRT: mean residence time, CL: clearance.

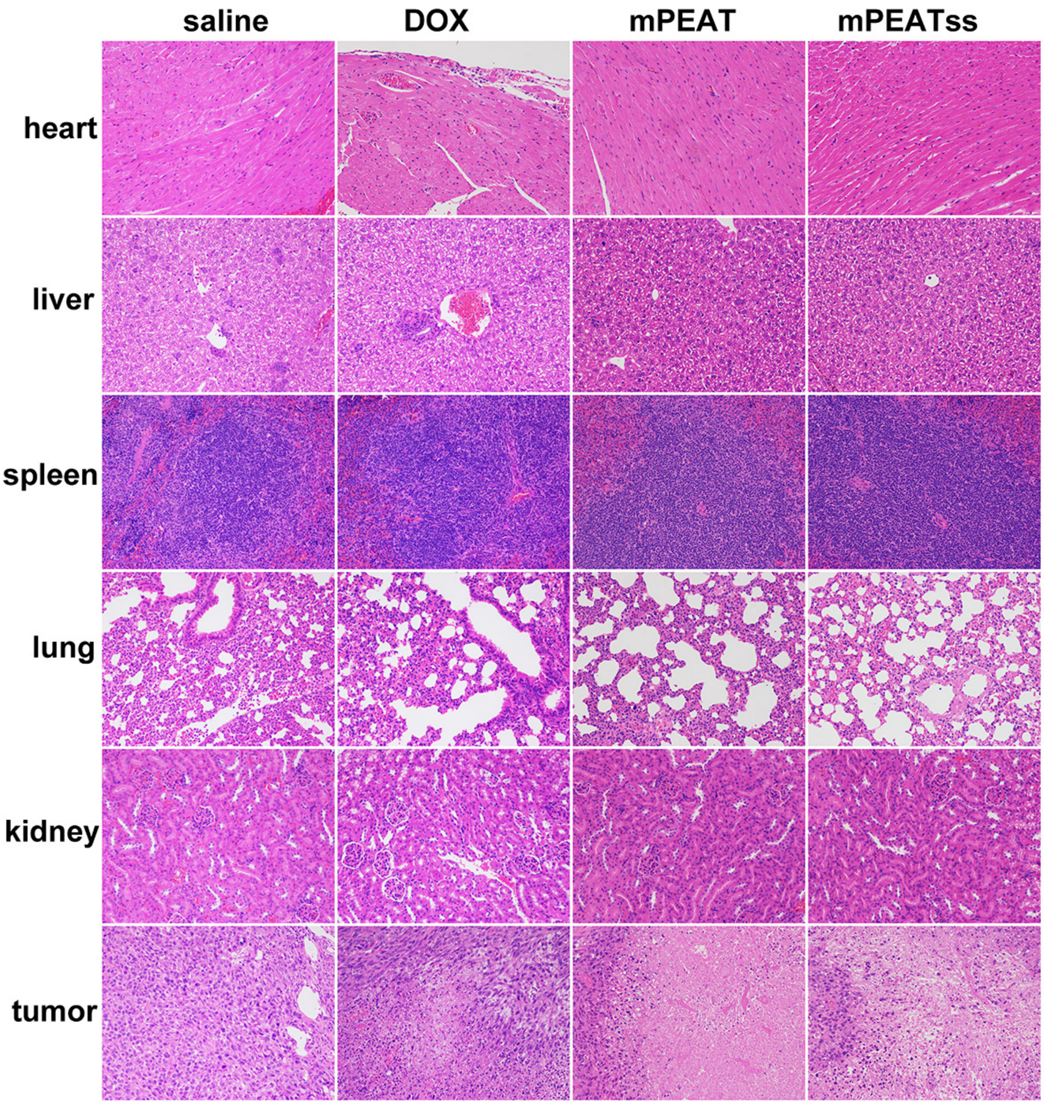

Figure S18. Images of H&E assays for tumors and major organs after different treatment after 21 d (all tissue: 200×).

References

1. Li D, Lu F, Wang J, et al. Amorphous metal-free room-temperature phosphorescent small molecules with multicolor photoluminescence via a host-guest and dual-emission strategy. J Am Chem Soc. 2018; 140: 1916-23.

2. Zhang Y, Jiang M, Han G, et al. Solvent effect and two-photon optical properties of triphenylamine-based donor−acceptor fluorophores. J. Phys. Chem. C 2015; 119: 27630-38.
